# On the encoding of natural music in computational models and human brains

**DOI:** 10.3389/fnins.2022.928841

**Published:** 2022-09-20

**Authors:** Seung-Goo Kim

**Affiliations:** Research Group Neurocognition of Music and Language, Max Planck Institute for Empirical Aesthetics, Frankfurt am Main, Germany

**Keywords:** system identification, naturalistic stimuli, computational model, natural music, artificial neural network, musical emotion

## Abstract

This article discusses recent developments and advances in the neuroscience of music to understand the nature of musical emotion. In particular, it highlights how system identification techniques and computational models of music have advanced our understanding of how the human brain processes the textures and structures of music and how the processed information evokes emotions. Musical models relate physical properties of stimuli to internal representations called features, and predictive models relate features to neural or behavioral responses and test their predictions against independent unseen data. The new frameworks do not require orthogonalized stimuli in controlled experiments to establish reproducible knowledge, which has opened up a new wave of naturalistic neuroscience. The current review focuses on how this trend has transformed the domain of the neuroscience of music.

## Introduction

Music is believed to have been a crucial part of all known societies from the very early days of the human species (Zatorre and Salimpoor, 2013). Bone flutes found near the Danube River in Germany suggest that the origin of music can be dated to about 40,000 years ago or more (Conard et al., 2009). Given that the emergence of *Homo sapiens* is believed to have emerged in Africa about 300,000 years ago (Hublin et al., 2017) and to have migrated from Africa to Eurasia around 60,000 years ago (Armitage et al., 2011), even earlier evidence of the musical traditions of humans may exist in Africa that is yet undiscovered (d’Errico et al., 2003). Furthermore, cross-cultural studies based on ethnographic texts and audio recordings provide empirical evidence that music appears in every society observed (e.g., Mehr et al., 2019); and this ubiquitous presence of music in human societies indicates music’s significant functions for humans. Zatorre and Salimpoor (2013) suggested the reason for music’s existence is that it allows for the expression and regulation of emotion and elicits pleasure. But the central question remains unsolved: How does music, a structured collection of abstract sounds, evoke such intensive emotions?

As neuroscientists understand it (Zatorre, 2005), music is processed by way of hierarchical pathways, potentially with feedbacks (Vuust et al., 2022), evoking emotions on multiple levels at different time scales (Juslin et al., 2013). According to the current understanding, waveforms of music are first transduced into neural activity throughout the auditory peripheral and central pathways, where spectral and temporal decompositions take place. The acoustic information is then believed to be transformed into musical events (e.g., notes, chords, beats). Musical structures (spectral and temporal relationships of musical events in the short and long terms; e.g., motifs, themes, tonalities, rhythms, tempi) can be recognized as statistical patterns depending on a listener’s prior experience (e.g., enculturation, training) or concepts based on explicit knowledge. While explanations based on predictive processing have suggested important mechanisms (Zatorre and Salimpoor, 2013; Vuust et al., 2022), how other kinds of information of music transform into emotions remains largely to be discovered.

In many neuroscientific studies, stimuli were created as simple “models” (or approximations) of complex stimulations in real-world environments while parametrizing variables of interest and orthogonalizing nuisance variables. The orthogonalization of stimuli provides a simple model of the world and linearizes the assumed effects of the variable of interest. For instance, in our own previous study (Kim et al., 2017), we investigated the effect of dissonant harmony on evoked emotional responses and individual preferences using functional magnetic resonance imaging (fMRI). To this end, we created “dissonant versions” of 30-s excerpts taken from various instrumental musical pieces by transposing the original excerpts by dissonant intervals (major second upward, and diminished fifth downward) and mixing them all down. These “dissonant versions” constantly produced dissonant harmony regardless of the tonal structures in the original pieces. The altered audio clips certainly evoked “unpleasantness” (i.e., all participants rated the Pleasantness Scale lower) and decreased blood oxygen level-dependent (BOLD) responses in the auditory pathway and other brain regions as compared with the responses to the original pieces. Because the dissonant stimuli were created without altering other acoustic aspects such as loudness, beats, rhythms, phrases, and so on, the design was optimal for investigating the linear effect of consonance (or dissonance) without concerns about the multicollinearity of acoustics. One problem, however, was that the observed effect (i.e., “people disliked the dissonant versions”) could not be generalized (i.e., “people dislike dissonant harmony”), because in real-world music such a dissonant harmony could nevertheless be perceived as “yet pleasant” when it is presented in different musical styles (Popescu et al., 2019). That is, although the effect of dissonant harmony was successfully found using the orthogonalized stimuli within the experiment, it remains unclear, unfortunately, how relevant the results are to our understanding of how harmony evokes various emotions beyond the experiment settings. Experimental approaches contrasting music vs. non-music stimuli can be seen as a “music-as-fixed-effect” fallacy, following the “language-as-fixed-effect” fallacy proposed by Clark (1973), who criticized the limited generalizability of simplistic, contrastive approaches in certain psycholinguistic research.

While not all controlled experiments suffer from limited validity, there might be difficulties stemming from their assumption that the human brain (or its behavior on average) is governed by simple, interpretable rules that can be discovered by cleverly isolated manipulations and can extrapolate to complex human behaviors (Nastase et al., 2020a). This misconception (or an arbitrary approximation) has been elegantly termed by Jolly and Chang (2019) as the “Flatland fallacy,” after Edwin Abbot’s famous short story (1884), which refers to a problem prevalent in psychology (and other cognitive sciences), wherein researchers misbelieve that “the parsimony offered by our low-dimensional theories reflects the reality of a much higher-dimensional problem” (Jolly and Chang (2019), p. 433). Whereas dimensionality reduction can be an efficient tool for many computational problems (i.e., “all models are wrong but some are useful” (Box, 1979), Jolly and Chang (2019) pointed out that the low-dimensional bias in cognitive sciences may be effected by “human” reasons (e.g., feelings of understanding, limitations of human cognitive capacity, cultural norms, communicating complexity) rather than computational reasons (e.g., predictive performance, computational cost). However, caution should also be taken against the humanly motivated high-dimensional bias (e.g., feelings of awe and excitement when met with an unprecedentedly large-scale artificial neural network, regardless of its efficiency).

To approach the complexity of real-world cognition and perception, naturalistic experiments are essential. The criticism against reductionism inherent in controlled experiments has a history in psychology (Brunswik, 1943; Gibson, 1978). In fact, even in neuroscience, the argument for naturalistic stimuli is not new (Barlow, 1961). One of the methodological arguments that has been discussed with regard to animal electrophysiological data (Rieke et al., 1995; Theunissen et al., 2000) has a striking resemblance to an assertion occurring in the recent discussions on human “naturalistic neuroimaging” (Sonkusare et al., 2019; Hamilton and Huth, 2020; Nastase et al., 2020a; Jääskeläinen et al., 2021): *the controlled stimulus may be too uninteresting for living animals, even for sensory neurons.* Moreover, the presumed linearity in sensory neurons may not hold given the non-linear responses to biologically salient stimuli (i.e., the sum of responses to subcomponents of a conspecific vocalization is smaller than the response to the whole vocalization in non-primary sensory neurons; Theunissen et al., 2000). Components that are uniquely responsive to speech and music (or their unique acoustic structures) found in human fMRI, electrocorticogram (ECoG), and electroencephalogram (EEG) data (Norman-Haignere et al., 2015, 2022; Zuk et al., 2020) also suggest a strong degree of non-linearity even in the human auditory cortex^1^.

The current review focuses on one of the distinguished recent advances in human neuroscience: the use of naturalistic stimuli supported by advanced computational models. Computational models provide us with not only physical descriptors but also information related to the underlying structures of natural images (Kay et al., 2008; Naselaris et al., 2009; Vu et al., 2011), natural videos (Nishimoto et al., 2011; Han et al., 2019), natural movies (Hasson et al., 2004, 2010; Hanke et al., 2014; Vodrahalli et al., 2018), and natural speech (Huth et al., 2012, 2016; Stephens et al., 2013; Mesgarani et al., 2014; Broderick et al., 2018; Nastase et al., 2020b), to name a few. In particular, this article attends to the recent investigations into the neural representation of natural music using computational models with a specific interest in musical emotion. For readers who are unfamiliar with model-based analyses, first predictive models, with mathematical descriptions, will be introduced in Section “Predictive modeling: From features to responses.” Then, along with application examples, music models will be reviewed in Section “Music modeling: From stimuli to features.” Finally, challenges and perspectives will be discussed in Section “Challenges and perspectives.”

## Predictive modeling: From features to responses

This section will guide readers from traditional linear models to non-linear models, highlighting how they relate to each other, in the context of predictive modeling. Please note that, in this section, we do not assume a specific neuroimaging modality. For the sake of discussion, let us assume that we have sufficient degrees of freedom in the temporal dimension given their respective inherent temporal dependencies and sampling rates. Exemplary applications to M/EEG and fMRI data, as will be provided throughout this section, demonstrate that a similar method can reveal different temporal scales of the brain activity of interest when applied to different data.

**FIGURE 1:**
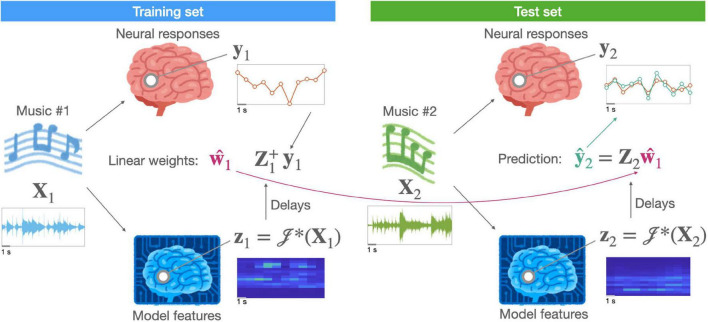
A schematic of a linearized encoding model. A hypothesized neural representation in the brain of music (**X**) is modeled by a computation model [i.e., a linearization function 𝒥*(⋅)] resulting in feature time-series (**z**). A linear relationship, described by a response function (**w**), between the delayed features (**Z**) and measured neural responses (**y**) is estimated using stimuli (e.g., music #1) in the training set. For independent stimuli (e.g., music #2) in the test set, the same computational model extracts features. Using them, the predictive performance of the hypothesized representation can be evaluated.

A model that predicts a response for a given stimulus based on an estimation how a stimulus is encoded in a system is called an encoding model. That is, the goal of an encoding model (see, e.g., Kay et al., 2008) can be understood as an identification of a transfer function that maps a given event (or a stimulus) in a physical space (e.g., time-points in audio signals) onto an evoked response in a measurement space (e.g., voxels in fMRI data or channels in M/EEG data). For instance,


(1) y=𝒥(X)+ε

where **y** is a vector of evoked physiological responses (as a function of either stimuli or time-points) of a certain measurement unit (e.g., a voxel or a channel); **X** is a matrix that describes physical properties of stimuli in the same time scale as **y**; and *𝒥*(⋅) is a transfer function (or a response function in the temporal domain) from stimuli to responses, which can be either linear or non-linear. The problem of estimating such transfer functions is traditionally known as *system identification* in various domains in engineering fields, including automatic control and signal processing (Zadeh, 1956; Keesman, 2011; Ljung et al., 2020). A popular approach to non-linearity in system identification, especially for naturalistic stimuli, is to approximate the system as a sequence of non-linear and linear transformations (Naselaris et al., 2011). That is, Equation 1 can be decomposed into a non-linear transform followed by a linear transform as:


(2) $$\text{y}={𝒥}^{*}\left(X\right)\text{w}+\varepsilon$$

where 𝒥*(⋅) is a non-linear function that maps stimuli (or time-points) from a physical space to a representational (or “feature”) space and **w** is a vector of weights for a linear transform that maps stimuli (or time-points) from a representational space onto a neural measurement space. The non-linear function 𝒥*(⋅), which afterwards enables a linear mapping, is known as *linearization* (for a general overview, see Wu et al., 2006). That is, a linearized encoding model can still capture non-linearity while using a linear mapping between the assumed (or hypothesized) features and evoked neural responses. Figure 1 illustrates a linearized encoding model.

For completeness, decoding models can also be seen as:


(3) $$\text{x}={𝒦}^{*}\left(\mathrm{YV}\right)+\varepsilon$$

where **x** is a vector of the properties of stimuli with respect to a certain physical property; **Y** is a matrix of evoked physiological responses, with each column corresponds to a measurement unit; **V** is a matrix of linear decoding weights that maps neural responses to features (each column corresponds to a feature); and 𝒦*(⋅) is a non-linear function that maps features back to physical properties. Note that a decoding model can be converted from an encoding model based on Bayes’ theorem, reflecting the prior of features (i.e., occurrence probability) in naturalistic stimuli (Naselaris et al., 2009). This is useful when the linearization function 𝒥*(⋅) is non-invertible (i.e., 𝒦*(⋅) cannot be found by the inverse of 𝒥*(⋅); e.g., the real absolute value function). See Naselaris et al. (2011) for a review of model-based decoding.

While the models need to be sufficiently flexible to capture the underlying transforms, the fitting (or learning) process is agonistic to the nature of variance (whether it is due to the underlying transforms or to independent noise). Therefore, a validation of an estimated model with unseen data is a crucial part of the system identification (Ljung, 2010). There are various schemes of validation (Hastie et al., 2009), but the common idea is to test the generalizability of an estimated model, which is based on one set of data (*training set*), with another set of unseen data (*test set*) with independent and identically distributed noise (i.e., the *i.i.d.* assumption). In practice, the “unseen data” can be created by holding out some part of the data from the model estimation (i.e., cross-validation). The partitions of training sets vs. test sets can be half vs. half (split-half), k-1 parts vs. 1 part (k-fold), or all samples but one vs. the held-out one (leave-one-out). For hyperparameter optimization, the whole dataset can be divided into three parts: a training set, a validation set, and a test set that respectively comprise roughly 50, 25, and 25% of the whole set (Hastie et al., 2009). The *validation set* is so named because it is used to validate the *hyperparameters^2^* . In some cases, the training set can be split into two parts: an inner-training set and a hyperparameter validation set (nested cross-validation). Taking a larger training set (i.e., *k* > 1) would make a test set smaller for a given dataset, which would increase the sample variance in the test set. Therefore, selecting the CV scheme (i.e., determining k) is also a matter of bias-variance tradeoff. In general, 5–10-folds could result in more stable test accuracies than the leave-one-out scheme (Varoquaux et al., 2017). Critically, the partitions should be carefully designed to avoid *information leakage* between the training sets and test sets (Kaufman et al., 2012; Glaser et al., 2020). In particular, functional time series in neuroscience typically exhibits strong spatial and temporal dependencies at different scales. Moreover, intra-/inter-subject repetitions of stimuli are highly prevalent in many experiment designs, which could allow the repeated stimuli to introduce high similarity between seemingly independent time points or subjects. For example, if one randomly assigns the individual samples of EEG data to training sets or test sets, autocorrelated noise would leak into other partitions. Cross-validation is a method to test the reproducibility of a given model. Therefore, it has been argued that a statistical inference should be focused on predictive performance rather than on observed sample statistics for a reproducible science (Yarkoni and Westfall, 2017; Varoquaux and Poldrack, 2019).

### Linear model

Many readers will be familiar with a multiple linear regression that models linear effects of experimental factors such as:


(4) y=Xβ+Zγ+ϵ

where **y** is a *n* × 1 vector of neural responses (of a certain measurement unit; e.g., a voxel) to a given set of stimuli, a *n* × *p* matrix **X** describes the variables (or properties) of interest of the stimuli over columns, a *n* × *q* matrix **Z** describes the nuisance variables over columns, and are *p* × 1 and *q* × 1 vectors of unknown coefficients, respectively, and *ϵ* is a *n* × 1 vector of Gaussian noise. Equation 4 can be solved using the ordinary least squares (OLS) method when the **X** and **Z** are orthogonally created by experimenters. If we replace **y** with a matrix of multiple units (e.g., channels, voxels) and *ϵ* with a matrix of uncorrelated Gaussian noise (i.e., without modeling inter-unit dependency), the linear model is called the general linear model (GLM), which is simply a set of identical univariate models applied to multiple units (i.e., “massive univariate testing” for large-scale units; Friston et al., 1994a). For the sake of simplicity, response variables in the following are written as vectors (i.e., univariate models). However, note that they can be easily extended to their massive-univariate equivalences by concatenating response and error terms.

In case sample-wise physiological measures are directly related to time-varying stimulus properties, a proper transfer function (or a response function in the temporal domain) from neural activity to physiological measurements needs to be determined. While such a function is known to spatially and temporally vary in non-trivial ways (Aguirre et al., 1998; Handwerker et al., 2004; Badillo et al., 2013; Taylor et al., 2018), if we assume such a transfer function *h*, Equation 4 can be rewritten (Friston et al., 1994b) as:


(5) $$\text{y}=h*\text{[}\begin{array}{cc} \text{X}\beta & Z\gamma \end{array}]+\varepsilon$$

where * denotes a convolution in the temporal domain and ε is a Gaussian noise with serial-correlation, which is typically non-zero in most of non-invasive measures. Note that all vectors and matrices are now defined for each sample (i.e., time-point) of the physiological measurement (i.e., the number of rows is the number of samples, *t*). Also, note that both **X** and **Z** describe stimulus properties so that they can be concatenated to apply the same convolution. Equation 5 can be solved using a variant (due to the serial-correlation) of OLS such as weighted least squares (WLS) (Friston et al., 2006; Poldrack et al., 2011).

Note that a linear model with OLS can also be used as a predictive model and be cross-validated. As long as the variables are orthogonal (or minimally intercorrelated), the OLS is an unbiased estimator for the training set. However, the estimates may not apply to the test set because the OLS will also fit the noise, together with the signal, in the training set. Regularization (see Section “Regularized linear methods”) can be useful in such cases. When a linear model is used as a predictive model, the inference will be on whether the prediction accuracy (e.g., Pearson correlation between prediction and observation) is above chance level as opposed to whether the estimated contrast (e.g., a difference between condition A vs. condition B) is above chance level.

### Reverse correlation

While Equation 5 can still model delayed processes between neural activity and measurement by a fixed physiological transfer function *h*, it cannot flexibly model delayed processes between stimulus and neural activity. A system identification method—also known as *reverse correlation* or *triggered correlation*, for the analysis averages stimuli based on response as opposed to averaging responses based on stimuli)—uses autocorrelation (or autocovariance) of the stimuli and the cross-correlation (or cross-covariance) between the stimuli and responses to capture the delayed responses in a response function (i.e., a linear filter) under the assumption of the system (i.e., a linear time-invariant [LTI] system). This was introduced in electrophysiology as a receptive field mapping technique by presenting white noise (instead of many narrow-band filtered signals) and estimating the frequency selectivity of individual neurons (Boer and Kuyper, 1968).

When we assume that a set of physical properties of stimuli that are relevant to the neural system of interest (i.e., often called *features*) {***f***_1_,***f***_2_,…,***f***_*p*_} are given—such as, e.g., narrow-band filtered acoustic energy of the presented white noise)—then Equation 5 can be rewritten as:


$$\text{y}=\left[\begin{array}{cccc} h_{1}*{\text{f}}_{1}\beta_{1} & h_{2}*{\text{f}}_{2}\beta_{2} & \cdots & h_{p}*{\text{f}}_{p}\beta_{p} \end{array}\right]+\varepsilon$$

where *h_i_* is a feature-specific convolutional kernel, or a transfer function, for the *i*-th feature; ***f***_*i*_ is a *n* × 1 vector of the *i*-th feature; and the effect size *β*_*i*_ is now simply a signed amplitude of the *h_i_*, which is in fact redundant. Therefore, we can further simplify:


(6) $$\text{y}=\left[\begin{array}{cccc} h_{1}*{\text{f}}_{1} & h_{2}*{\text{f}}_{2} & \cdots & h_{p}*{\text{f}}_{p} \end{array}\right]+\varepsilon$$

In the case of discrete signals, the convolution above (Equation 6) can be rewritten as a multiplication of delayed features **G** and finite impulse response (FIR) functions **w** defined over finite delays {*l*_1_, *l*_2_, …, *l*_*d*_} as:


(7) y=Gw+ε

where


$$\text{G}=\left[\begin{array}{c} {\text{F}}_{1}\;{\text{F}}_{2}\;\ldots \;{\text{F}}_{p} \end{array}\right]\in {\mathbb{R}}^{n\times pd},$$

$$\;F_{\text{i}}=\left[\begin{array}{ccc} f_{i}(t_{1}-l_{1}) & \cdots & f_{i}(t_{1}-l_{d})\\ \vdots & \ddots & \vdots \\ f_{i}(t_{n}-l_{1}) & \cdots & f_{i}(t_{n}-l_{d}) \end{array}\right]\in {\mathbb{R}}^{n\;\times \;d}$$

i.e., [**F**_i_]_*j*,*k*_ = *f*_*i*_(*t*_*j*_ − *l*_*k*_), *f*_*i*_(*t*) is the element of the *i*-th feature vector at the timepoint *t*, **y** is a *n* × 1 vector of neural responses from the timepoint *t*_1_ to *t_n_*, **w** = [**u**_1_ **u**_2_ ⋯ **u**_p_]^T^ ∈ ℝ^*pd* × 1^, and ***u***_*i*_ is a *d* × 1 vector of a discrete response function to estimate. If the delays {*l*_1_, *l*_2_, …, *l*_*d*_} are adjacent to each other in the sample space (i.e., *l*_*j* + 1_ − *l*_*j*_ = *t*_*i* + 1_ − *t*_*i*_), **F**_i_ is a *n* × *d* rectangular Toeplitz matrix (i.e., *t*_*i*_ − *l*_*j*_ = *t*_*i* + 1_ − *l*_*j* + 1_). Note that the FIR model (Equation 7) has been used to fit a physiological response function (e.g., a hemodynamic function) to the data in controlled experiments (Henson et al., 2001). In reverse correlation, the FIR function is used to further include the transform from stimulus to neural activity in addition to the transform from neural activity to non-invasive measurement (e.g., fMRI image intensity or scalp-EEG potential).

If **G** is fully ranked—i.e., features are not correlated, such as white noise—then we can use the OLS to estimate **w**:


(8) $$\hat{\text{w}}={\left({\text{G}}^{\text{T}}\text{G}\right)}^{-1}{\text{G}}^{\text{T}}\text{y}={\text{G}}^{+}\text{y}$$

where the hat operator $\hat{\cdot }$ denotes an estimation; ⋅^T^ denotes a matrix transposition; ⋅^−1^ denotes a matrix inversion; and ⋅^+^ denotes Moore-Penrose pseudoinversion^3^. Note that the OLS solution minimizes the prediction error (i.e., loss function) on the training set itself given as:


$$\text{L}\left(w;y\right)={∥{\left(\text{y}-\text{Gw}\right)}^{T}\left(\text{y}-\text{Gw}\right)∥}_{2}={∥\text{y}-\text{Gw}∥}_{2}^{2}$$

For a discrete timeseries **a** ∈ ℝ^*n*^, an autocovariance matrix for *d* lags is given by **A**^T^**A** ∈ ℝ*^d × d^* where **A** is a *n* × *d* Toeplitz matrix. With another timeseries **b** ∈ ℝ^*n*^, a cross-covariance matrix of **a** and **b** is given by **A**^T^**B** ∈ ℝ*^d × p^* where **A** and **B** are *m* × *d* and *m* × *p* Toeplitz matrices, respectively. Therefore, Equation 8 can be rewritten as:


(9) w^=(GTG)-1GTy=CGG-1cGy

where **C_GG_** is a *pd* × *pd* autocovariance matrix and **c_Gy_** is a *pd* × 1 cross-covariance vector since **y** is not delayed. Note that this expression highlights the fact that the method *decorrelates* the stimulus-response cross-covariance with the autocovariance of the stimulus itself. If the features do not have any autocovariance structures—for example, if a single predictor is given by white noise—then the sample autocovariance matrix of the predictors can be very close to the identity matrix: **C_GG_** ≈ **I**.

Note that the reverse correlation method itself does not require regularization when the stimulus is well-behaving Gaussian. Therefore, for linear systems, or for systems that can be well approximated by linear models, the OLS solution is sufficient. However, this is not the case in many real-world systems including the human brain (and even the auditory neurons as discussed earlier).

### Regularized linear methods

In general, regularization serves two purposes: one is to avoid overfitting, even for a model with a single predictor, by penalizing overly complex models; and the other is to deal with strong multicollinearity present in the predictors (i.e., an ill-posed inverse problem). Note that even for a single-feature model (i.e., *p* = 1 in Equation 6), multicollinearity may exist across delayed features (i.e., columns of **G** in Equation 7) if serial-correlation is present in the feature. Now that the **C_GG_** in Equation 10 can be very different from **I** and non-invertible, it is necessary to introduce regularization to make it invertible. Tikhonov regularization (Tikhonov, 1943; Tikhonov et al., 1995) is a general solution with a regularization matrix **Λ** ∈ ℝ*^pd × pd^*:


(10) $${\hat{\text{w}}}^{{}^{*}}\left(\Lambda \right)={\left({\text{G}}^{\text{T}}\text{G}+\Lambda \right)}^{-1}{\text{G}}^{\text{T}}\text{y}$$

where **Λ** = **Γ**^T^**Γ** when the loss function to minimize is defined with the L2 norm penalty as:


$$\text{L}\left(w;y,\Gamma \right)={∥\text{y}-\text{Gw}∥}_{2}^{2}+{∥\Gamma \text{w}∥}_{2}^{2}$$

Ridge regression is a special case of Tikhonov regularization where **Λ** = *λ***I** and *λ* is a regularization scalar (Hoerl and Kennard, 1970):


(11) $${\hat{\text{w}}}^{{}^{*}}\left(\lambda \right)={\left({\text{G}}^{\text{T}}\text{G}+\lambda \text{I}\right)}^{-1}{\text{G}}^{\text{T}}\text{y}={\left({\text{C}}_{\mathrm{GG}}+\lambda \text{I}\right)}^{-1}{\text{c}}_{\mathrm{Gy}}$$

Note that the ridge solution (and its prediction performance) is a function of the regularization. The regularization controls the flexibility of the model, which impacts the bias (i.e., the expected distance between “true” parameters and estimates *across multiple experiments*) and variance (i.e., the spread of estimates across multiple experiments) of the solutions in opposite ways. For example, estimates from an extremely rigid model, such as the 0-th order model that always returns a constant, will be highly biased but have no variance. In other words, it will be wrong but in a very consistent fashion. On the other extreme, a flexible model will be minimally biased (i.e., accurate on average), but largely varied. That is, it can be sometimes very accurate, but it can also be very wrong, depending on the realization of random noise. The “best” regularization depends on the “true” structure of the system (Ljung et al., 2020), which is unknown. Therefore, in practice, the regularization is “optimized” to balance the tradeoff between bias and variance for specific datasets. Several optimization methods have been used for fMRI and M/EEG data acquired while listening to natural speech and music: e.g., ridge tracing (Santoro et al., 2014; Moerel et al., 2018), bootstrapping (Huth et al., 2016), and nested cross-validation (Daube et al., 2019).

From a Bayesian perspective, the Tikhonov regularization can be seen as multivariate normal priors on the “distribution of weights” (Nunez-Elizalde et al., 2019) as:


(12) $$\text{w}∼{𝒩}_{pd}\left(0,\lambda^{-2}\Sigma \right)$$

where *𝒩_pd_* is a *pd*-dimensional multivariate normal distribution and Σ ∈ ℝ*^pd × pd^* is the positive definite prior covariance matrix, and *λ* is a scalar regularization parameter. The ridge regularization can be seen as a special case of spherical priors (i.e., **Σ** = **I**) whereas other forms of regularization can be seen as non-spherical priors (Nunez-Elizalde et al., 2019). The maximum likelihood solution of the problem can be found in a closed form as a Tikhonov solution (Nunez-Elizalde et al., 2019):


$${\hat{\text{w}}}_{T}\left(\lambda ,\Gamma \right)={\left({\text{X}}^{\text{T}}\text{X}+\lambda^{2}\Gamma^{\text{T}}\Gamma \right)}^{-1}{\text{X}}^{\text{T}}\text{y}$$

where **Σ**^−1^ = **ΓΓ**^T^. This is equal to a ridge solution when **Γ** = **I** (thus, **Γ**^T^**Γ** = **I** = **Σ**). The Tikhonov solution can be simplified (Nunez-Elizalde et al., 2019) by a linear transform such that:


$$\text{A}=\text{X}\Gamma^{-1}$$

Then, a ridge solution with **A** is given as:


$${\hat{\text{w}}}_{A}\left(\lambda \right)={\left({\text{A}}^{\text{T}}\text{A}+\lambda^{2}\text{I}\right)}^{-1}{\text{A}}^{\text{T}}\text{y}$$

The estimates can be projected back into the original space, which finally gives us the Tikhonov solution:


$${\hat{\text{w}}}_{T}\left(\lambda ,\Gamma \right)=\Gamma^{-1}{\hat{\text{w}}}_{A}\left(\lambda \right)$$

Now, the prior matrix **Σ** (or the inverse transform matrix **Γ**) can be based on stimulus models (e.g., a semantic embedding), physiological models (e.g., hemodynamic response function [HRF]), or appreciation of different scales of features, i.e., independently regularizing features or feature spaces, because the globally optimal regularization could be suboptimal for individual features (i.e., “banded ridge”; Nunez-Elizalde et al., 2019). In fact, the last usage is widely known as “multi-penalty ridge.” It was first proposed in the original publication of ridge regression (Hoerl and Kennard, 1970) in sections 5 (p. 63) and 7 (p. 65), where a regularization parameter (“*k_i_*”) is found for each column of the orthogonalized design matrix (i.e., canonical variates). The multi-penalty ridge can be seen as a general case of ridge where the Tikhonov regularization matrix (Equation 10) is given as:


$$\Lambda =\left[\begin{array}{ccc} \lambda_{1}{\text{I}}_{1} & \cdots & 0_{k}\\ \vdots & \ddots & \vdots \\ 0_{1} & \cdots & \lambda_{1}{\text{I}}_{k} \end{array}\right]$$

where *λ*_*i*_ is a scalar hyperparameter for the *i*-th feature space, **I**_*i*_ ∈ ℝ^*p*_*i*_*d* × *p*_*i*_*d*^ is an identity matrix for the *i*-th feature space with *p*_*i*_ as the number of columns of the *i*-th feature space, and **0**_*i*_ ∈ ℝ^*p*_*i*_*d × p*_*i*_*d*^ is a zero square matrix. Recent studies optimized a regularization parameter for each feature space to perform model comparisons without *over-regularization*, i.e., suboptimal regularization for specific features; see, e.g., Daube et al. (2019) and Sohoglu and Davis (2020).

However, the optimization of *p* hyperparameter (i.e., determination of regularization parameters) via grid-search would have a complexity that is proportional to *j^k^* for *j* grid points and *k* hyperparameters, which rapidly makes the optimization intractable. There are fast algorithms for multi-penalty ridge problems that are inspired by the original formulation (Hoerl and Kennard, 1970) and where the design matrix is first orthogonalized to reduce the number of necessary hyperparameters [see, e.g., van de Wiel et al. (2021) for applications on the “large *p*, small *n*” genomic data].

Besides the ridge penalty, other types of penalty terms are also commonly used such as lasso (i.e., L1 norm penalty; Tibshirani, 1996), which has been used for naturalistic speech MEG data (Brodbeck et al., 2018a,b), and elastic net (i.e., both of L1 and L2 penalties; Zou and Hastie, 2005).

### Non-linear kernel methods

So far, we have discussed linear methods. However, there are various methods for handling non-linearity, even in the field of traditional machine learning and statistical learning. Kernel-based machine learning methods, such as the *support vector machine* (SVM; Boser et al., 1992) can be seen as a linearization of features in the sense that it provides non-linear transformation of original predictors into a high-dimensional feature space where a linear fit can be useful (Bishop and Nasrabadi, 2006). The idea is that some non-linear-looking problems can be seen as linear in a higher-dimensional space. Let such a mapping from a lower-dimensional original predictor space to a higher-dimensional feature space (i.e., *features space mapping*) be ϕ : ℝ^*p*^→ℝ^*q*^ where *p* < *q*. Equation 6 then can be defined in the *feature* space:


(13) $$\text{y}={\left[\begin{array}{cc} \begin{array}{cc} \phi {\left({\text{g}}_{1}\right)}^{\text{T}} & \phi {\left({\text{g}}_{2}\right)}^{\text{T}} \end{array} & \begin{array}{cc} \ldots & \phi {\left({\text{g}}_{n}\right)}^{\text{T}} \end{array} \end{array}\right]}^{\text{T}}\text{w}+\varepsilon$$

where ***g***_*i*_ is the *i*-th row vector in the matrix **G** ∈ ℝ*^n × pd^* from Equation 7.

Now the problem becomes how we can find the feature space mapping in unknown high (theoretically infinite) dimensions. Fortunately, instead of explicitly finding this mapping to unknown dimensions, it has been shown that the prediction can nonetheless be made without knowing the mapping itself, but rather with a kernel function *k*, which is an inner product of transformed features: *k*(***g***_1_, ***g***_2_) = ⟨ϕ (***g***_1_), ϕ (***g***_2_)⟩ (Bishop and Nasrabadi, 2006). A prediction on the new datapoint ***g***_*x*_ with a regularization *λ* can be given without explicitly knowing the mapping ϕ(⋅) as:


(14) $$\hat{\text{y}}\left({\text{g}}_{x}\right)=\phi \left({\text{g}}_{x}\right)\hat{\text{w}}=k\left({\text{g}}_{x}\right){\left({\text{K}}_{\text{t}}+\text{λ}{\text{I}}_{n}\right)}^{-1}{\text{y}}_{t}$$

where the Gram matrix [**K**_t_]_*i*,*j*_ = *k*(***g***_*i*_, ***g***_*j*_) is defined over all *n* datapoints in the training set, **I**_*n*_ ∈ ℝ*^n × n^*, **y**_*t*_ = [*y*_1_ *y*_2_ ⋯ *y*_*n*_]^T^. This substitution (also known as *kernel trick*) of the unknown mapping with the Gram matrix that is defined between datapoints makes the problem tractable. The kernel function can be constructed with non-linear basis functions such as the *p*-th order polynomial with a constant term c: $k\left({\text{x}}_{\text{i}},{\text{x}}_{\text{j}}\right)={\left({\text{x}}_{\text{i}}^{\text{T}}{\text{x}}_{\text{j}}+c\right)}^{p}$, Gaussian basis function: *k*(**x**_i_, **x**_j_) = exp (− ∥**x**_i_ − **x**_j_∥^2^ /2σ^2^), or the radial basis function: *k*(**x**_i_, **x**_j_) = exp(−*γ* ∥ **x**_i_ − **x**_j_ ∥^2^) (Bishop and Nasrabadi, 2006). The hyperparameters of the kernels can be validated through cross-validation in practice (Chu et al., 2011). Kernel regression (alibeit with linear kernels) has also been used for auditory encoding models (De Angelis et al., 2018; Erb et al., 2019; Rutten et al., 2019).

Related to the kernel trick, *representational similarity analysis* (RSA) compares a kernel of the brain with a kernel of a reference model (Kriegeskorte et al., 2008). RSA does not attempt to explicitly identify the transfer functions of a system (i.e., the first-order isomorphism between the physical properties and representations in the brain), but it can query whether systems share similar non-linear mappings of the identical set of stimuli (i.e., the second-order isomorphism between representations in different systems), which establishes important foundations for understanding the human brain as a non-linear system.

### Neural network methods

Recently, with increased computational capacity under Moore’s law and large-scale (i.e., petabytes) data (Sun et al., 2017), modern artificial neural network (ANN) models have outperformed traditional models (including traditional ANNs) and are reaching human-level performances in many tasks such as semantic visual recognition (Donahue et al., 2014), language generation (Floridi and Chiriatti, 2020), and even a specific scientific discovery activity (Jumper et al., 2021). In general, modern ANN models, also known as deep neural networks (DNNs), have multiple (“deep”) layers of units (e.g., perceptrons) and include iterative adaptive processes, which allow a model to update (or “learn”) its parameters (or weights) based on the binary labels (or continuous values) given by humans (supervised learning) or real data (unsupervised learning). A perceptron can be seen as a linear binary classifier (Bishop and Nasrabadi, 2006) as:


(15) $$f\left({\text{x}}_{i}\right)=\text{sign}\left({\text{x}}_{i}\text{w}\right)$$

where $\text{sign}\left(\text{a}\right)=\{\begin{array}{c} \text{​}+1,\;a\geq 0\\ -1,\;a<0 \end{array}$, **x**_*i*_ is a 1 × *p* vector of features of the *i*-th instance (e.g., a stimulus), **w** is a *p* × 1 vector of weights. As seen earlier (Equation 13), a non-linear transformation ϕ(⋅) can be introduced:


(16) $$f\left({\text{x}}_{i}\right)=\text{sign}\left(\phi \left({\text{x}}_{i}\right)\text{w}\right)$$

By combining such simple units, a network can be very flexible. For example, a prediction from a two-layer network can be expressed (Bishop and Nasrabadi, 2006) as:


(17) $$\hat{y}\left({\text{x}}_{i},\omega \right)=\sigma \left(h\left({\text{x}}_{i}{\text{w}}^{\left(1\right)}\right){\text{w}}^{\left(2\right)}\right)$$

where σ(⋅) is a sigmoid function, *h*(⋅) is a hyperbolic tangent function, **w**^(*i*)^ is a vector of weights at the *i*-th layer, and $\omega =\left[\begin{array}{c} w^{\left(1\right)}\\ w^{\left(2\right)} \end{array}\right]$ is a vector of all weights. (i.e., a set of all weight vectors). The loss function of this network can be defined as a sum-of-squares error function:


(18) $$\text{L}\left(\omega ;ℳ\right)=\sum_{x_{i}\in ℳ}{∥\hat{y}\left({\text{x}}_{i},\omega \right)-y_{i}∥}^{2}$$

where ℳ = {**x**_1_, **x**_2_, …, **x**_*n*_} is a training set and *y*_*i*_ is the true response (or label) of *x*_i_. Given the non-linearity, the loss function cannot be analytically minimized. However, the loss function can still guide the model to adjust weights to reduce errors for the next example. This process is called backpropagation (Amari, 1967; Werbos, 1974, 1994). One of the widely used approaches is called *gradient descent* optimization (Bishop and Nasrabadi, 2006). The idea is that, even without knowing the loss function analytically, one can still empirically minimize it by perturbating each weight and figuring out which direction in the weight space would decrease, or at least not increase, the loss function. More formally, one can compute partial derivative with respect to changes of individual weight for the *i*-th data point: $\frac{\partial \text{L}\left(\omega ;{\text{x}}_{\text{i}}\right)}{\partial w_{j}^{\left(i\right)}}$ where $w_{j}^{\left(i\right)}$ is the *j*-th weight in the *i*-th layer. A vector of partial derivatives is called a *gradient*. For a function *f* that maps a *n*-dimensional real vector **x** = [*x*_1_ *x*_2_ ⋯ *x*_*n*_] to a real scalar (i.e., *f* : ℝ^*n*^ → ℝ), the gradient of *f* is defined as: $\nabla f\left(x\right):=\left[\begin{array}{cc} \begin{array}{cc} \frac{\partial f}{\partial x_{1}} & \frac{\partial f}{\partial x_{2}} \end{array} & \begin{array}{cc} \cdots & \frac{\partial f}{\partial x_{n}} \end{array} \end{array}\right]$. Thus, a gradient is a “direction” vector (in the weight space) that maximizes a given function. Therefore, we would like to update our weight by subtracting the gradient but by a small magnitude. That is, given the *i*-th training data point, the next weight can be expressed as:


(19) $$\omega_{i+1}=\omega_{i}-\gamma \nabla \text{L}\left(\omega_{i};{\text{x}}_{i}\right)$$

where ω_*i* + 1_ is the weights learned from the *i*-th data point, γ is a control parameter called a *learning rate*.

DNN models learn weights in various forms of architectures consisting of multiple well-known structures with several practical modifications. For example, a convolutional neural network (CNN; LeCun et al., 1989) is a multilayer architecture that includes convolutions in the input space (e.g., with respect to horizontal and vertical axes in 2-D images; with respect to time dimension in the audio waveform) to exploit a topographical organization (i.e., local dependency) of the data. This can be seen as a strong prior (i.e., “geometric knowledge about the task”; LeCun et al., 1989, p. 550) that completely disconnects some connections (Goodfellow et al., 2016).

Another fundamental architecture known as a recurrent neural network (RNN) (Rumelhart et al., 1986) was developed to learn structures in sequential data such as language (e.g., word sequences). In this network, a node gets inputs from a node at a previous time point as well as the current stimulus at the current time point, returns outputs for the current time point, and feeds an input to a node at the next time point.

Recently, DNN models have been widely used in finding a non-linear relationship in human neuroimaging data. However, an improvement by using non-linear models over linear models requires a high SNR and/or a very large sample size as Gaussian noise can linearize the decision boundary (Schulz et al., 2020). Except for a few consortia (e.g., UK Biobank, Human Connectome Project), large-scale functional data, especially with naturalistic stimuli, are scarce in comparison to behavioral data (e.g., crowdsourced tagging data for millions of songs). Therefore, in many applications for naturalistic stimuli, DNN models are trained to replicate large-scale human behavioral data, then models’ representations (i.e., linearized features) are related to smaller-scale human neural data via regularized linear models (Agrawal et al., 2014; Güçlü and van Gerven, 2015; Güçlü et al., 2016; Caucheteux and King, 2022) or representational similarity analysis (Khaligh-Razavi and Kriegeskorte, 2014; Kell et al., 2018). In particular, CNN models mimicked human auditory behaviors (e.g., pitch [F0] perception, word recognition, musical genre recognition) and neural responses (Kell et al., 2018; Schulz et al., 2020), arguing for a representational gradient across the superior temporal gyrus/sulcus (Güçlü et al., 2016). While the CNN is considered to be one of the greatest achievements of neuromorphic engineering (i.e., a system that is inspired by the hierarchical structure of the sensory system of brains, performing perceptual tasks at a near-human-level) and has shown a partial convergence with neural representations in various sensory modalities (Agrawal et al., 2014; Cadieu et al., 2014; Khaligh-Razavi and Kriegeskorte, 2014; Yamins et al., 2014; Güçlü and van Gerven, 2015; Kell et al., 2018), their implication (i.e., whether they can be accepted as evidence for a mechanical model of a human sensory system) is still under debate (see Section “Challenges and perspectives”).

## Music modeling: From stimuli to features

This section discusses recent endeavors in modeling the structure of music. From the perspective of encoding models, a computational music model can be seen as a hypothesized linearization and an inference on the model performance (and additional contributions of individual sets of features) would be equivalent to effect testing in controlled experiments. Models at various levels and their applications to natural music will be reviewed.

### Auditory models

Models based on psychophysics and electrophysiology have been developed to simulate the neural activity of the auditory pathway (Chi et al., 1999; Klein et al., 2000). Among various formulations, a MATLAB implementation, namely Neural Systems Laboratory (NSL) tools^4^ (Chi et al., 2005) was created based on electrophysiological findings. In the first stage, the *auditory spectrogram* is computed, which is a time-frequency representation of given sound signals. The basilar membrane filter bank is modeled by bandpass filters. The outputs are further processed accounting for various non-linear transforms through the auditory nerves. Then a lateral inhibition in the cochlear nucleus is simulated by the first-order derivative across frequency channels. This cascade model can be described as follows:

(1) cochlear filter bank:


$$y_{c}\left(t,f\right)=s{\left(t\right)}^{*}h\left(t;f\right)$$

where *y*_*c*_(*t*, *f*) is a cochlear output at time *t* and frequency channel *f* (often referred to as [simulated] *cochleogram*), *s*(*t*) is a signal at time *t*, * is a convolution operator in the temporal domain, *h*(*t*; *f*) is a response function of the *f*-th frequency channel (i.e., a membrane filter),

(2) auditory nerve:


$$y_{a}\left(t,f\right)=g{\left(\partial_{t}y_{c}\left(t,f\right)\right)}^{*}w\left(t\right),$$

where *y*_*a*_(*t*, *f*) is an auditory-nerve output, *g*(⋅) is a non-linear compression (i.e., gain), ∂_*t*_ denotes partial differential with respect to time as a high-pass filter, *w*(*t*) is a low-pass filter that mimics the decrease of phase-locking above 2 kHz,

(3) cochlear nucleus:


$$y_{l}\left(t,f\right)=\max\left(\partial_{f}y_{a}\left(t,f\right),0\right)$$

where *y*_*l*_(*t, f*) is a cochlear-nucleus output, ∂_*f*_ denotes partial differential with respect to frequency to mimic the lateral inhibition in the cochlear nucleus, max (⋅, 0) is a half-wave positive rectifier,

(4) and, finally, midbrain:


(20) $$y_{m}\left(t,f\right)=y_{l}{\left(t,f\right)}^{*}\mu \left(t;\tau \right)$$

where *y*_*m*_(*t*, *f*) is the midbrain (final) output, and *μ*(*t*; τ) is a short-time (τ = 8 ms) integration window (for further loss of phase-locking in the midbrain).

Then, in the second stage, the *cortical representation* is computed by a 2-D convolution of an spectrotemporal respective field (STRF) filter bank and the auditory spectrogram. For a specific “cell” that is sensitive to a specific combination of spectral and temporal modulations (i.e., “spatial” components in the spectrogram) and a direction (downward or upward), the cortical representation *z* is given as:


(21) $$z_{c⇓\left(⇑\right)}\left(t,f;\omega_{c},{\text{Ω}}_{c},\theta_{c},\phi_{c}\right)=y_{m}\left(t,f\right)⊗{\text{STRF}}_{c⇓\left(⇑\right)}$$

where *c* ⇓ (⇑) denotes a downward (or upward) cell *c*, ω_*c*_ is the temporal modulation rate (i.e., ripple velocity; in Hz) of the cell *c*, Ω_*c*_ is the spectral modulation scale (i.e., ripple density; in cycles/octave), θ_*c*_ is the rate phase, ϕ_*c*_ is the scale phase, and ⊗ denotes 2-D convolution. STRF_*c*⇓(⇑)_ is defined as the real part of the product of two complex functions describing temporal modulations (ripples along the time axis in the auditory spectrogram) and spectral modulations (ripples along the frequency axis) as:


$${\text{STRF}}_{c⇓\left(⇑\right)}=ℛ\left\{h_{IRT}^{{(}^{*})}\left(t;\omega_{c},\theta_{c}\right)\cdot h_{IRS}\left(t;{\text{Ω}}_{c},\phi_{c}\right)\right\}$$

where ℛ{⋅} denotes the real part, *h*_*IRS*_ and *h*_*IRT*_ denotes complex impulse response functions for temporal modulation and spectral modulation, respectively, and the superscripted * denotes the complex conjugate, which allows for differentiation of downward and upward tone sweeps. Because the third and fourth quadrants can be constructed using complex conjugates of the STRFs in the first two quadrants, we only consider those two quadrants (1st: positive rate, positive scale, downward; 2nd: negative rate, positive scale, upward). Further details can be found in Chi et al. (2005).

While this model has been widely used for various short (1–2 s) naturalistic audio clips and speech (Moerel et al., 2013; Santoro et al., 2014; Khalighinejad et al., 2019; Sohoglu and Davis, 2020), applications to naturalistic music remain relatively sparse: ECoG data over the left frontotemporal areas while a highly trained pianist playing 2-min classical pieces with and without auditory feedback (Martin et al., 2017), ECoG data over bilateral frontotemporal regions from 29 patients while listening to a 3-min song with vocals (Bellier et al., 2022), and whole-brain fMRI data while listening to five-hundred-forty 15-s excerpts from the GTZAN (G. Tzanetakis and P. Cook) Musical Genre Dataset (Nakai et al., 2021). These studies consistently demonstrated that the auditory models (the auditory and cortical representations) successfully predicted neural responses to natural music in the primary and non-primary auditory cortices. A preliminary report suggested redundant information is encoded in non-auditory regions such as sensory-motor cortices and the inferior frontal gyrus (Bellier et al., 2022).

### Music information retrieval models

Music information retrieval (MIR) is an interdisciplinary field that has emerged with the arrival of the electronic music distribution systems (e.g., *Napster* in 1999, *iTunes* in 2001, and *Spotify* in 2006) (Aucouturier and Bigand, 2012). Therefore, its main focus is to develop technologies that are useful for such services including searching, organizing, accessing, and processing digitized music signals and related data. Compared to music psychology, which focuses more on processes via simplistic examples, the MIR focuses more on the “end-to-end” results (i.e., linking physical characterizations of music signals and population behaviors, possibly in the music market) (Aucouturier and Bigand, 2012). Nonetheless, the MIR field has discovered multiple acoustic features that are predictive of “perceived emotions” in music (Yang and Chen, 2011), which are not necessarily experienced by listeners, but are recognizable. These findings motivated psychologists and neuroscientists to investigate whether this functional (end-to-end) relationship implies anything for the internal processes in listeners, even at a population level, because this may have relevance to “experienced emotions.”

Traditional MIR models, as opposed to recent ANNs, tend to have a couple hundred (counting all subcomponents) “hand-crafted” features. Some of the features in MIRtoolbox (Lartillot and Toiviainen, 2007) are briefly explained here for a discrete signed signal *s*(*x*) of *t* timepoints:

- Root-mean-square (RMS) envelope: $E=\sqrt{\frac{1}{t}\sum_{x=1}^{t}s{\left(x\right)}^{2}}$ for global energy, $E\left(w\right)=\sqrt{\frac{1}{n}\sum_{x=1}^{n}w{\left(x\right)}^{2}}$ with a windowed signal w(x) of *n* (< *t*) timepoints for local energy.

- Zero-crossing rate: z = $\frac{1}{t-1}\sum_{x=1}^{t-1}𝒥\left(s\left(x\right)s\left(x-1\right)\right)$ where $ℐ\;(y)=\{\begin{array}{c} 1,\;y<0\\ 0,\;y\geq 0 \end{array}$, which can also be locally calculated for a given windowed signal. In a simplistic case (e.g., a sine wave), this can be used to estimate the fundamental frequency. However, more generally, it describes some aspects of timbral quality rather than pitch.

- Spectrogram: **S**(*w*, *f*) = ***f***(*k*) *H*(*k*; *f*) with ***f***(k) the magnitude of a discrete Fourier transform (i.e., fast Fourier transform) of a given windowed signal $\text{f}\left(k\right)=|\sum_{m=0}^{n-1}w\left(x\right)\text{exp}\left(-2\text{π}ikm/n\right)|,k=0,..,n/2$ and *H*(*k*; *f*) is a transfer function of a given filter bank for a characteristic frequency in a linear scale (Hz). Various filter banks based on behavioral psychophysical experiments have been used: e.g., Mel-scale and Bark-scale. The logarithm of magnitude is more often used. This describes acoustic energy decomposed in frequency bands.

- Cepstral coefficient: $\text{C}\left(w,k\right)=\sum_{f=0}^{p-1}\text{S}\left(w,f\right)\;\cos\;\left[\frac{\pi }{n}\left(f+\frac{1}{2}\right)k\right],$ *k* = 0, …, *p* − 1 for a spectrogram defined over *p* frequency bands. Because in most of natural stimuli, spectrograms have high spectral dependency (magnitudes in adjacent channels are similar), discrete cosine transform is used to further compress and orthogonalize the spectrogram.

- Spectral flux: *L*(*w*_*i*_, *w*_*i*−1_; **S**) = ∥**S**(*w*_*i*_) − **S**(*w*_*i*−1_)∥_2_ with **S**(*w*_*i*_) a spectrum (or a cepstrum) for the *i*-th window. It could be more sensitive to frequency-specific energy changes compared to the RMS. Given that natural musical pieces could have widely various spectra (i.e., unnormalized), in some cases, this metric could mainly reflect the spectral density.

- Spectral centroid: $N\left(w;S\right)=\sum_{f=0}^{F-1}K\left(f\right)\text{S}\left(w,f\right)/$∑*f* = 0^*F*−1^**S**(*w*, *f*) for a given filter bank *S* that constructs the spectrogram **S**(*w*, *f*) over *p* frequency bins with characteristic frequencies *K*(*f*). That is, a weighted average of characteristic frequencies *K*(*f*) of where the weights are normalized magnitudes.

- Key clarity: *K* (**S**) = *max*_*i*∈*𝒦*_ corr (ψ_*i*_, θ(**S**)) where 𝒦 is a set of all 12 major and 12 minor keys, ψ_*i*_ is the tonal stability profile of the *i*-th key (Krumhansl and Shepard, 1979), and θ(⋅) is a chromagram of a given spectrogram (a power spectrum for 12 pitch classes based on the standard 440-Hz tuning). The key similarity (i.e., corr(ψ_*i*_, θ(**S**))) can be used to find a most possible key. The maximal correlation with any key is used as a measure of the key clarity.

- Pulse clarity: $P\left(E\right)=\max_{l\in L}\sum_{x=0}^{n-1}E\left(x\right)E\left(x-l\right)$ where L is a set of lags. That is, maximal autocorrelation of the envelope at any lag is used as a measure of pulse clarity.

It should be noted once again that the primary goal of the MIR features is to describe audio contents at a low computational cost (e.g., real-time computation for automatic music identification services such as Shazam), rather than to describe psychological correlates of acoustic properties (Aucouturier and Bigand, 2012). Therefore, the names of the MIR features are only to serve practical purposes (i.e., they make it easier for human users to remember than numerical indices) and are at best suggestive, but do not necessarily allow for psychological interpretations. One example could be “key clarity.” Because it is a maximal correlation with any possible key, it rather describes how clear a key is to an algorithm based on Krumhansl’s profiles and cross-correlation than how it sounds to general human listeners. This discrepancy may be negligible when estimating a key based on a chromagram averaged across a whole excerpt as done in the original algorithm (Gómez, 2006). However, when the metric is calculated for short (1–3 s) frames, the discrepancy can be non-trivial. In principle, any clearly presented triad can have a high “key clarity” value even if the chord is distant from the dominant key along the circle of fifths. That is, even if a chord is tonally unstable, disturbing the overall tonality (e.g., Db major triad in C major key; i.e., the famous Neapolitan chord), its “key clarity” could be as high as C major triad in C major key (i.e., tonic). A similar discrepancy could exist for other metrics, such as “pulse clarity,” when they are computed for short frames. Thus, readers who wish to better understand the nature of MIR features are strongly encouraged to study the documents provided by the developers of respective implementations. Only for intuitive illustrations, readers can find exemplary audio clips with minimal or maximal values of the listed MIRtoolbox features from 985 intact songs (Sturm, 2013) in GTZAN Musical Genre Dataset (Tzanetakis and Cook, 2002)^5^ in Supplementary Files 1, 2.

One of the most exciting characteristics of the MIR models lies in the open-source principle: many well-maintained packages are freely available online: e.g., librosa (McFee et al., 2015)^6^, MIRtoolbox (Lartillot and Toiviainen, 2007)^7^, Essentia (Bogdanov et al., 2013)^8^, and more^9^. This has allowed for a rapid adaptation of MIR features in predicting neural responses to natural music in EEG data (Cong et al., 2012, 2013; Sturm et al., 2015, 2017; Stober, 2017; Kaneshiro et al., 2020; Wang et al., 2020; Leahy et al., 2021), intracranial EEG data (Sturm et al., 2014; Omigie et al., 2020), and fMRI data (Alluri et al., 2012, 2013; Toiviainen et al., 2014; Casey, 2017; Hoefle et al., 2018). These studies consistently revealed that the MIR features extract relevant information that is predictive of ongoing neural activity during naturalistic music listening. Some features seem to be more reliable than others in predicting fMRI signals. In particular, when a lasso regression with a fixed canonical hemodynamic function was used as a predictive model (Alluri et al., 2012; Burunat et al., 2016), short-term features that are based on a 25-ms window (mostly describing spectral contents and their short-term dynamics) showed greater reliability than long-term features that are based on 3-s window (“key clarity” and “pulse clarity”) (Burunat et al., 2016). The “long-term” measures attempted to capture higher-level perceptions, such as tonal center or meter recognition. While the long-term features could still be useful for differentiating musical pieces (thereby decoding perceived emotions and musical genres from music signals), the studies show that localizing the neural correlates of musical percepts in time can be difficult. In a recent study (Leahy et al., 2021), differential encoding of meters and beats (e.g., for a 4/4 time signature, strong-weak-middle-weak vs. four beats without accents) was detected in human EEG data using an automated beat-tracking algorithm (McFee et al., 2015). This suggests that an improvement of linearization may lead to applications of encoding models beyond low-level sensory processing.

It would also be worth mentioning, although it slightly deviates from the main focus of the current review, that the MIR models can be used to objectively describe acoustic features of natural musical stimuli in *controlled* experiments (see, e.g., Whitehead and Armony, 2018). Given that the MIR features would be more sensitive than traditionally used aggregated metrics such as overall RMS, loudness, and spectra, the MIR models can be used as tools to control or match nuisance variability in stimuli. Alternatively, biological models, more explicit modeling of the neural activation throughout the auditory pathway, can also be used to match global statistics (e.g., the first four moments [mean, variance, skewness, and kurtosis] of the cortical representations) (Norman-Haignere and McDermott, 2018).

Another usage of the MIR model is an automatic annotation of musical events to perform analyses comparing neural responses among different events or correlating an aggregated metric of neural responses with the extracted features: e.g., event-related potential in EEG data (Poikonen et al., 2016a,b), dynamic functional connectivity analysis in fMRI data (Singer et al., 2016; Toiviainen et al., 2020), and inter-subject synchronization in fMRI data (Trost et al., 2015; Sachs et al., 2020). However, while traditional methods are computationally efficient and readily available, their performance needs to be taken with caution. In a recent EEG study (Haumann et al., 2021), the MIRtoolbox missed 41.6–45.0% (based on either RMS or spectral flux) of perceivable onsets that were manually detected by an expert rater (i.e., a musicologist), which deteriorated the following EEG analyses based on the automatically extracted onsets. Recent models based on neural networks such as Madmom (Eyben et al., 2010)^10^ are known to outperform traditional onset extraction models (∼7% error rate when tested on datasets including music with percussive sessions), which encourages researchers not to be restricted by the traditional models.

More recently, pretrained DNN models have been used to extract their embeddings in new MIR research (Lee and Nam, 2017; Grekow, 2021; Grollmisch et al., 2021). In particular, VGGish [Visual Geometry Group-ish] (Hershey et al., 2017) and Open-L3 [Look, Listen, and Learn more] (Cramer et al., 2019) are CNNs that were developed to generate text labels for given short (∼1 s) audio signals. Both models are pre-trained on large-scale video data (i.e., 60 million AudioSet clips and 8 million YouTube clips) exploiting the correspondence between image and audio data in video sources. In a sense, the networks effectively learn the second-order isomorphism between the image frames and the audio spectrograms. The possibility of a transfer learning of these networks to MIR tasks has been investigated. Koh and Dubnov (2021) extracted audio embeddings using the VGGish and Open-L3 models, then created shallow classifiers (e.g., SVM, Naïve Bayes, and Random Forest) to decode emotional classes (e.g., four quadrants on the Arousal-Valence space or six emotional categories). The CNN embeddings outperformed (32–88% in decoding accuracy) the conventional MIR descriptor (e.g., Mel-Frequency Cepstral Coefficient [MFCC] as a baseline; 31–46%), demonstrating their relevance to music emotion recognition at the excerpt level. In visual domain, the CNN embeddings of images have been found to be related to affective ratings and fMRI responses in univariate and multivariate fashions (Kragel et al., 2019; Horikawa et al., 2020; Koide-Majima et al., 2020), suggesting distributed representations of emotion-specific features (i.e., high-order statistical descriptors of physical properties that are differentially associated with diverse emotions) in the human cortical networks (Sievers et al., 2021). Taken together, DNN embeddings of music are expected to serve as effective predictors for the MIR tasks and the neural encoding analysis.

### Computational musicological models

In computational musicology, music is often modeled as a sequence of symbols (e.g., a sequence of notes forms a melody of one part, a sequence of chords forms harmonic progressions and tonality). While this approach ignores multiple “unscored” variability in music signals including timbre, dynamics, and tempo, which are known to be very relevant to emotional responses and associated neural activity (Chapin et al., 2010; Bresin and Friberg, 2011; Trochidis and Bigand, 2013), this approach enables *scalable* analyses on musical structures (Rohrmeier and Cross, 2008; Moss et al., 2019; Rohrmeier, 2020; Hentschel et al., 2021). That is, once symbolic representations are collected, an analysis can be scaled up to a large volume of corpora using computers, a task that would take decades or more for human experts (musicologists) to complete. Moreover, neuroscientific studies based on this approach have investigated how the musical structures form anticipations in spectral and temporal domains in listeners’ minds and how they evoke emotional responses via suspended fulfilment or betrayal of such anticipations (for a review of the “predictive coding of music” model, see Vuust et al., 2022).

One successful model, called Information Dynamics Of Music (IDyOM; Pearce, 2005), is a variant of the *n*-gram model (Shannon, 1948) based on a combinatory n-gram model called Prediction by Partial Match (PPM; Cleary and Witten, 1984). For a given sequence with *k* events *s* = {*e*_1_, *e*_2_, …, *e*_*k*_} where *e*_*i*_ denotes the *i*-th event in the sequence and all discrete events are from a finite set (*e*_*i*_ ∈ ℳ) and a sub-sequence from the *i*-th event to the *j*-th event is denoted as $s_{i}^{j}=\left\{e_{i},e_{i+1},\ldots ,e_{j}\right\}$, the conditional probability to observe an event *e*_*i*_ after observing a preceding sequence $s_{1}^{i-1}$ can be approximated by the (*n*-1)-th order Markov (i.e., *n*-gram) model, whose maximum likelihood (ML) estimate is given as:


(22) $$\mathrm{Pr}\left(e_{i}|s_{1}^{i-1}\right)\approx {\hat{\mathrm{Pr}}}_{n}\left(e_{i}|s_{1}^{i-1}\right)=\left\{ \begin{array}{cc} 1/\left|ℳ\right|,\;c\left(e_{i}|s_{\left(i-n\right)+1}^{i-1}\right)=0\; & \\ \frac{c\left(e_{i}|s_{\left(i-n\right)+1}^{i-1}\right)}{\sum_{d\in ℳ}c\left(d|s_{\left(i-n\right)+1}^{i-1}\right)},\;otherwise\; & \end{array}\right.$$

where |⋅| denotes the number of elements of a set (i.e., cardinality); *c*(*e*|*s*) is the number of counts (in the training sets or corpora) of an event *e* after a sequence *s* from a given training set; and *d* is any event from the finite set ℳ. When the transition appears for the first time, a fixed probability based on the size of the set ℳ can be defined (Pearce, 2005). Put differently, the model estimates the probability by counting transitions in the training set. To allow the model to flexibly learn musical styles across compositions and the local context within each composition, long-term models (counting transitions only across corpora) and short-term models (counting transitions only within the current composition) were combined (Pearce, 2005; Harrison and Pearce, 2018) as in the PPM model (Cleary and Witten, 1984). A Common LISP implementation of the model is available online^11^. This particular model has been developed to predict the pitch and duration of coming notes in monopoly melodies. This allows us to compute the uncertainty of the context (i.e., entropy) and the negative log likelihood of a certain event (i.e., the change of entropy, also known as information content or surprisal) and has successfully predicted neural data (EEG and ECoG from different participants) while listening to MIDI-generated piano melodies extracted from J. S. Bach’s Chorales via encoding models (Di Liberto et al., 2020) in line with a previously reported association between conditional probability and evoked neural responses demonstrated with brief orthogonalized stimuli (Koelsch and Jentschke, 2010; Kim et al., 2011). A behavioral study using MIDI-generated flute melodies from classical compositions revealed that the information content had an inverted U-shaped effect (i.e., the *“Wundt”* effect; Wundt, 1874) on mean liking (i.e., an intermediate level of surprisal was preferred over extreme levels), and this effect was modulated by the uncertainty of contexts (Gold et al., 2019). Using a more generalized variant of PPM with memory decay over time, a similar antisymmetric pattern of behavioral responses (preferences for low-uncertainty/high-surprisal or for high-uncertainty/low-surprisal pairs) was reported in an fMRI experiment using chord sequences extracted from McGill Billboard corpus (Cheung et al., 2019), where the interaction between the uncertainty and information content was parametrically localized in clusters over the amygdala/hippocampal complex and medial auditory cortices.

A more commonly used model in symbolic analyses (Mor et al., 2020) is a hidden Markov model (HMM; Baum and Petrie, 1966). The HMM models conditional probabilities between latent (non-observable) states rather than between surface (observable) states, which allows for non-local dependencies and underlying (non-observable) structures in musical compositions to be explicitly expressed (Pearce and Rohrmeier, 2018). Various kinds of HMM models have been used to model different musical structures including melody, rhythm, and harmony (Raphael and Stoddard, 2004; Mavromatis, 2009). The HMM models showed greater prediction performance than *n*-gram models for certain musical structures (e.g., chord progressions in a jazz corpus; Rohrmeier and Graepel, 2012). However, the application of the HMM in related studies has been done mainly as a classifier that decodes entire musical pieces or genres from EEG signals (Kaur et al., 2017; Ntalampiras and Potamitis, 2019) rather than as a predictive model of musical structures.

### Generative neural network models

It has been argued that a prominent method for understanding the statistical structures of natural data is to create a system that can synthesize data *de novo* (Odena et al., 2017). Even if Richard Feynman’s famous dictum (“What I cannot create, I do not understand”) is true, only its contrapositive (“What I understand, I can create”) is also true, not its inversion (“What I can create, I understand”). That is, creating synthetic data would be necessary, but not sufficient, for understanding data. Having said that, various DNN models for music generation have been developed both in the audio and symbolic domains demonstrating the improvements in building such a synthetic system. While the successful performance of such models (e.g., synthetic speech and music that are physically and perceptually similar to real data) does not entail that the model “understands” the natural structures, it has drawn great attention in various fields (see Section “Interpretations of high-dimensional models”).

As an example of a symbolic CNN model, MidiNet is a modified deep convolutional generative adversarial network (Yang et al., 2017), where a generator network *G* generates artificial data to “fool” a discriminator network *D*, which distinguishes real data from generated data. For a given *t* × *p* binary matrix **X** that encodes onsets of notes over *p* pitch classes and *t* time steps (quantized beats) and a random noise vector **z**, the objective function of the generative adversarial networks (GANs) is given as:


(23) $$\min_{\text{G}}\max_{D}V\left(D,G\right)={𝔼}_{X∼p_{data}\left(X\right)}\log D\left(X\right)+{𝔼}_{z∼p_{z}\left(z\right)}\text{log}\left(1-D\left(G\left(z\right)\right)\right)$$

where **X** ∼ *p*_*data*_(**X**) denotes the sampling from real data and **z** ∼ *p*_*z*_(**z**) denotes the sampling from a random distribution. The output of the generator $G\left(\cdot \right)=\hat{\text{X}}$ is a generated “score” with a random input, and the output of the discriminator *D*(⋅) is in [0, 1] such that 1 for real data and 0 for generated data. Over iterations, the *G* learns weights that minimize this function (e.g., *D*(*G*(**z**)) ≈ 1) while the *D* learns weights that maximizes it (e.g., *D*(*G*(**z**)) ≈ 0). Further details including stabilization and conditioning can be found in the publication (Yang et al., 2017).

Amongst symbolic RNN models, Melody RNN by Google Magenta^12^ and Folk-RNN^13^ are well known. Both use long short-term memory (LSTM) units (Hochreiter and Schmidhuber, 1997) to model non-local dependencies. In particular, Folk-RNN is an RNN with three LSTM layers of 512 units (Sturm et al., 2016a), of which a vast number of parameters (over 5.5 million) were trained over 23,000 transcriptions of traditional tunes from Ireland and the UK (with over 4 million tokens [discrete classes of music notation including meter, mode, pitch, and duration]).

In the audio domain, WaveNet^14^ by DeepMind is a CNN model that operates on individual audio samples (i.e., amplitude at each time-point) at 16 kHz (Oord et al., 2016). Unlike image-CNN models, all connections between layers were causal (i.e., only nodes that process previous and current, but not following, time steps are connected to a node in a higher layer), so that the temporal order of the underlying structures in the audio data can be preserved.

Another well-known architecture for generative models is a variational autoencoder (VAE; Kingma and Welling, 2013), which introduced a variational Bayesian approach to “non-linear principal component analysis” (Kramer, 1991). The VAE models comprise an encoder, which finds efficient latent representations that are continuous (i.e., being able to be interpolated) and interpretable (i.e., the Euclidian distance between two classes in the latent space reflects “semantic” distance between two classes), and a decoder that generates new data from samples in the latent space. MusicVAE by Google Magenta is a symbolic model (Roberts et al., 2018) and Jukebox^15^ by OpenAI is an audio model (Dhariwal et al., 2020). In general, multiple time scales or hierarchical structures are used to capture non-local dependency in musical structures. In particular, Jukebox first learns latent representations of audio samples at three temporal scales (i.e., a sequence of 8, 32, 128 audio samples at 44,100 Hz) using VAE, then quantizes learned patterns into discrete tokens. Then, a transformer is trained on the tokens with a context covering ∼23 s at the longest temporal scale. In a recent study (Castellon et al., 2021), the middle layer of the transformer (4,800 features), which describes the 23-s audio patterns, showed a better performance in predicting arousal and valence ratings (66.9%) as compared to other models including a MFCC model (37.2%) and a CNN model (58.5%).

Recently, VAE models were shown to decode highly realistic face images from fMRI data (VanRullen and Reddy, 2019; Dado et al., 2022). In those studies, latent representations of face images (i.e., a high-dimensional vector representing an image) were extracted using the encoders of the VAE models, then these were used as predictors for fMRI responses (as a weighted linear sum of the vectors) to the corresponding face images. Using this linear model, latent representations were predicted for unseen images using fMRI responses. Finally, using the decoders of the VAE models, face images were reconstructed from the estimated latent representations. These experiments suggest a high similarity in information structures that the generative models and the brain extract from natural images. However, whether this can be generalized to the music domain, in particular in relation to musical emotion, remains to be investigated.

## Challenges and perspectives

### Stimuli for predictive modeling

So far, we have discussed why naturalistic stimuli are needed. To summarize, (1) the non-linearity of the neural system renders responses to controlled stimuli weak, (2) the external validity of a controlled experiment can be highly limited when applied to a non-linear system, and (3) recent developments of computational models of natural data provide testable models of non-linear transforms. However, there are also clear disadvantages of natural stimuli for experiments, which keep researchers inclined to orthogonalized stimuli (Hamilton and Huth, 2020): (1) multicollinearity, (2) over/under-representation, and (3) domain-specificity. As shown in Equation 9, the estimate of an encoding model reflects the serial-correlation of features and the multicollinearity among features, which would be minimized when white noise is used as a stimulus. In natural music, similarly to many other natural stimuli, various properties are often highly correlated (e.g., the strong correlation between pitch and onset density in a Western corpus; Broze and Huron, 2013); many features follow the power-law distributions (e.g., pitch, chord transition, and timbre in classical corpora, contemporary Western popular music, and cross-cultural corpora of folk songs; Serrà et al., 2012; Mehr et al., 2019; Moss et al., 2019); and the patterns of multicollinearity are variable across musical styles, cultures, and time (Broze and Huron, 2013; White, 2014; Pearce, 2018). Furthermore, a small (*n* = 10–20) set of exemplary stimuli typically used in neuroimaging experiments could over-represent certain covariance patterns that are different from the population of natural stimuli.

A brute-force approach to mitigating this problem would be to sample a massive set of stimuli. For example, the Natural Scenes Dataset (Allen et al., 2022) comprises ∼38 h/subject of 7-T fMRI data collected over ∼40 sessions watching 10,000 pictures of natural scenes with three repetitions, resulting in a total of 70,566 unique pictures from 8 subjects. Interestingly, but unsurprisingly, the DNN models predicted brain responses worse than did a traditional model (i.e., Gabor wavelet) with small samples (e.g., when trained on < 1,000 pictures with three repetitions) in some subjects, but were far better with more samples (e.g., > 3,000 pictures with three repetitions). If such massive-stimulus (i.e., “deeply-sampled”) high-quality datasets with naturalistic music are shared as open-source resources, it would foster rapid advances in the field (Poldrack and Gorgolewski, 2014).

While the large-scale stimuli data are necessary to investigate how the biases and variance in small sets impact estimates, there can be specific cases where only limited stimuli can be used (e.g., pediatric or elderly populations, epileptic patients undergoing neurosurgery). In such cases, a stimuli selection can be made in order to reduce multicollinearity and alleviate over/under-representation. Insofar as such a selection could degrade the ecological validity of the experiment to some extent, an optimal tradeoff should be carefully determined.

### Neural measurements

Encoding and decoding models can suffer from excessive noise in data, which is typically very high in most of the non-invasive measurements of neural signals. For instance, the fractional signal change induced by neuronal activity is estimated to be 1–2% at 3 T (Uludağ et al., 2009) and a simulation study found the SNR of M/EEG signals between –30 and –20 dB (Goldenholz et al., 2009). Especially for long, complex naturalistic stimuli, it can be difficult (or could violate assumptions such as non-familiarity of presented stimuli) to repeat the identical stimuli many times, which is a commonly used denoising technique (e.g., event-related potentials), whereby many trials with identical stimuli are averaged to cancel out non-stimulus-locked activities. Alternatively, there are denoising techniques that have been used for resting-state fMRI data, which is also, in a sense, single-trial data. The methods for suppressing non-physiological noise (e.g., spin-history artifacts due to head motions) and non-neural noise (e.g., fluctuations due to cardiac pulses and respirations) in fMRI data (see Caballero-Gaudes and Reynolds, 2017 for a review) include: RETROICOR (image-based retrospective correction; Hu et al., 1995), CompCor (component based noise correction; Behzadi et al., 2007), and ICA-AROMA (ICA-based automatic removal of motion artifacts; Pruim et al., 2015); these methods have been widely used for resting-state data and are applicable for naturalistic experiments as well. Task-based denoising techniques, such as GLMdenoise (Kay et al., 2013) and GLMsingle (Prince et al., 2021), that extract principal components that are not related to the experiment design has been used for encoding models with naturalistic stimuli and found to be beneficial for multivariate pattern analysis (Charest et al., 2018). Recently, a DNN-based interpolation method that reconstructs fMRI volumes while removing independent noise has been developed (Lecoq et al., 2021).

Multi-echo fMRI sequence (Posse et al., 1999) with a dedicated denoising technique (i.e., ME-ICA, multi-echo imaging with spatial independent component analysis; Kundu et al., 2013) has been suggested to separate BOLD signals (i.e., physiological) from other signals (e.g., non-physiological artifacts such as head motion, thermal noise from subjects and electronics, device imperfection) by exploiting a linear dependency of the BOLD effect on echo times. Although multi-echo fMRI requires a larger voxel and/or a longer time of repetition (TR) than the standard fMRI sequence for multiple readouts [e.g., ∼4-mm iso-voxel and TR of 2 s in multi-echo fMRI (Kundu et al., 2013) as compared to 2-mm iso-voxel and TR of 2 s in conventional single-echo fMRI, i.e., ∼8 times larger in volume], simultaneous multi-slice (also known as multi-band) acceleration techniques are expected to make the spatial and temporal resolutions of the multi-echo fMRI comparable to single-echo fMRI (Kundu et al., 2017).

Besides the lab-based neural measurements that require gigantic machines such as fMRI and MEG, wearable and portable EEG systems have been developed and already used in various naturalistic paradigms. For instance, wireless EEGs were used for hyper-scanning two pianists performing a piano duet (Zamm et al., 2020); and wireless systems were used to simultaneously collect physiological activity (e.g., electrocardiogram, facial muscle electromyogram, respiration, heart rates) from whole audiences (∼40 participants per concert) attending live string quintet performances (Czepiel et al., 2021; Merrill et al., 2021; Tschacher et al., 2021). In particular, the feasibility of a wireless in-ear EEG system has drawn considerable attention (Looney et al., 2014; Bleichner and Emkes, 2020; Nithya and Ramesh, 2020). A built-in EEG system in everyday devices (e.g., wireless in-ear headphones) might open a new possibility of collecting neural data from millions of people while they listen to their favorite music in their day-to-day lives.

### Interpretations of high-dimensional models

It may be broadly accepted that DNN models can serve, with caution, as functional models of some aspects of human cognition (notions such as ‘a DNN model processes similar information as humans do for certain tasks for some aspects’). But whether they can serve as a mechanistic model, even for a particular domain, (ideas such as ‘weights of a certain layer in a DNN model correspond to effective connectivity between neurons in a human brain’) remains under debate (Kay, 2018). In fact, this point has created heated discussions in the field of cognitive neurosciences (Kriegeskorte, 2015; Kay, 2018; Cichy and Kaiser, 2019; Kell and McDermott, 2019; Kriegeskorte and Golan, 2019; Lindsay, 2021; Pulvermüller et al., 2021). In particular, it has been pointed out that evidence and counterevidence should be examined in an unbiased fashion (Guest and Martin, 2021).

A promising approach has been suggested that involves investigating, instead of the first-order isomorphism between the physical properties of an object and its representation in a system, the second-order isomorphism between representations of multiple objects in multiple systems (Kriegeskorte et al., 2008). Investigating the representations in systems is consistent with efforts to “understand” (or interpret) the high-dimensional models. Various techniques for the interpretation of the DNN models have also been vigorously discussed and developed (Sturm et al., 2016b; Montavon et al., 2018; Sturm, 2018; Keshishian et al., 2020). In particular, Kay (2018) summarized three practical approaches for deepening our understanding of high-dimensional models: (1) we can observe the model’s behaviors to stimuli (i.e., mapping to the lower, intuitive space), (2) we can manipulate the models (i.e., perturbing parameters and observing performance chances), and (3) we can model a model (i.e., creating a simpler form that approximates the model’s simulated behaviors). Efforts to understand how models process musical information would be critical to deepening our intuitions as to how the human brain processes musical information.

## Conclusion

The current review discussed predictive models used for encoding and decoding analyses and music models that capture acoustics and underlying structures. In particular, predictive models have introduced a reproducible form of cognitive neuroscience and computational models have provided us with quantitative metrics that can be compared with neural representations of natural music. Novel data with large-scale stimuli and high-dimensional models are expected to allow us to better handle the non-linearity of the musical brain.

## Author contributions

The author confirms being the sole contributor of this work and has approved it for publication.

## Footnotes

1 There is a “third way” proposing controlled but naturalistic stimuli generated by sophisticated generative models such as generative adversarial networks (GANs). See Goetschalckx et al. (2021).

2 In other literature (e.g., Varoquaux et al., 2017),, a “validation set” can refer to what we call a “test set” inasmuch as it validates *model weights*.

3 In Penrose (1955), Corollary 1 states that the general solution of a linear model **y** = **Xb** is $\hat{\text{b}}={\text{X}}^{+}\text{y}+\left(\text{I}-{\text{X}}^{+}\text{X}\right)\text{a}$ where ***a*** is arbitrary (notations are rewritten for consistency with the current article). For real-valued, full-ranked X, **X** ^+^ = (**X** ^T^**X**)^−1^**X** ^T^. Thus, **X** ^+^ **X** = (**X** ^T^**X**)^−1^**X** ^T^**X** = **I**, leaving only the first term of the solution: $\hat{\text{b}}={\text{X}}^{+}\text{y}$.

4
http://nsl.isr.umd.edu/downloads.html

5
https://www.kaggle.com/datasets/andradaolteanu/gtzan-dataset-music-genre-classification

6
https://librosa.org

7
https://www.jyu.fi/hytk/fi/laitokset/mutku/en/research/materials/mirtoolbox

8
http://essentia.upf.edu/

9
https://www.ismir.net/resources/software-tools/

10
https://madmom.readthedocs.io/

11
http://mtpearce.github.io/idyom/

12
https://github.com/magenta/magenta/tree/main/magenta/models/melody_rnn

13
https://github.com/IraKorshunova/folk-rnn

14
https://www.deepmind.com/blog/wavenet-a-generative-model-for-raw-audio

15
https://openai.com/blog/jukebox/
