# Supplementary material for: On the encoding of natural music in computational models and human brains
Source: Front Neurosci. 2022 Sep 20;16:928841. doi: 10.3389/fnins.2022.928841 (PMC9531138; doi:10.3389/fnins.2022.928841)

## Slide 1
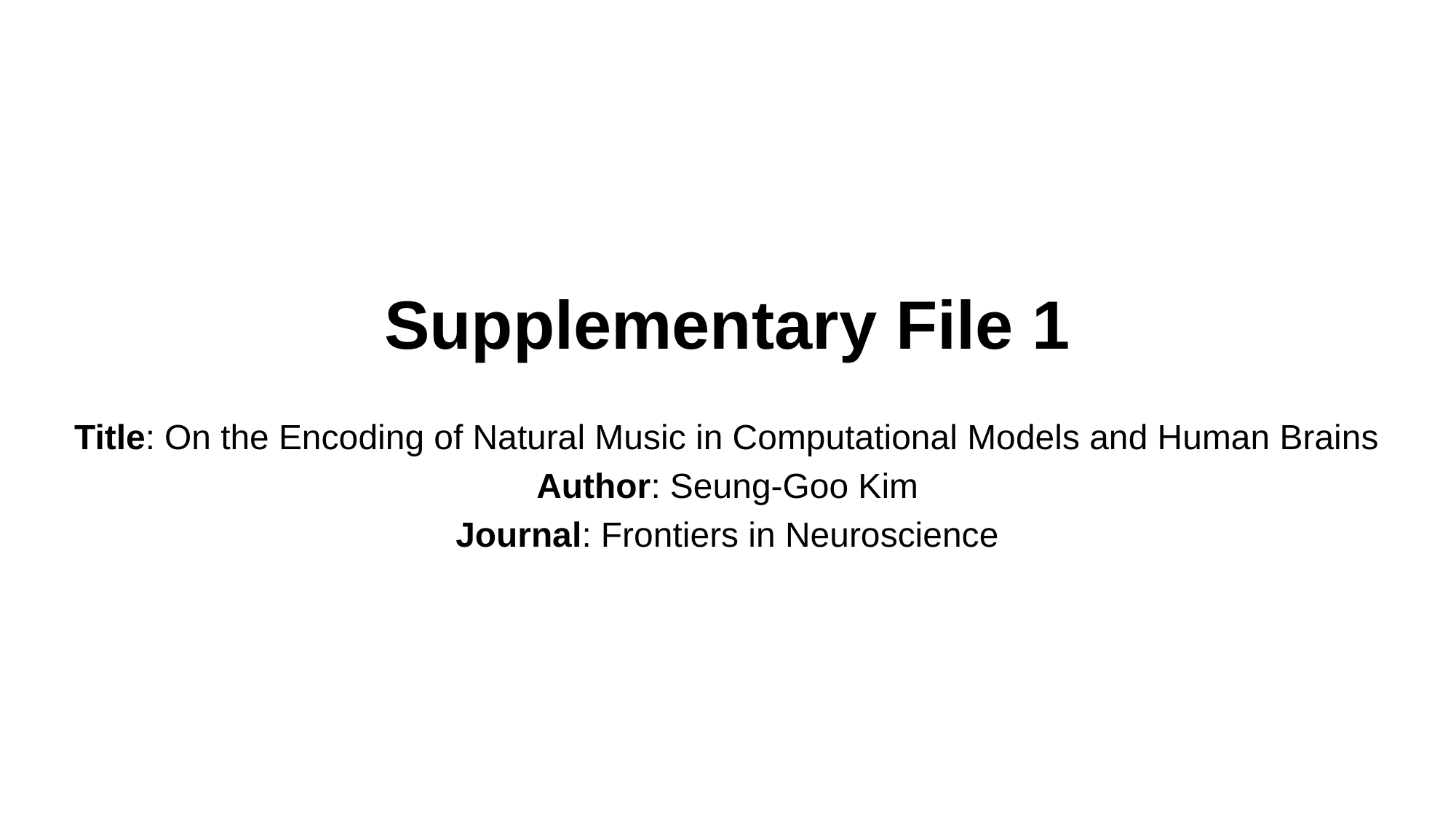

# Supplementary File 1
Title: On the Encoding of Natural Music in Computational Models and Human Brains
Author: Seung-Goo Kim
Journal: Frontiers in Neuroscience

## Slide 2
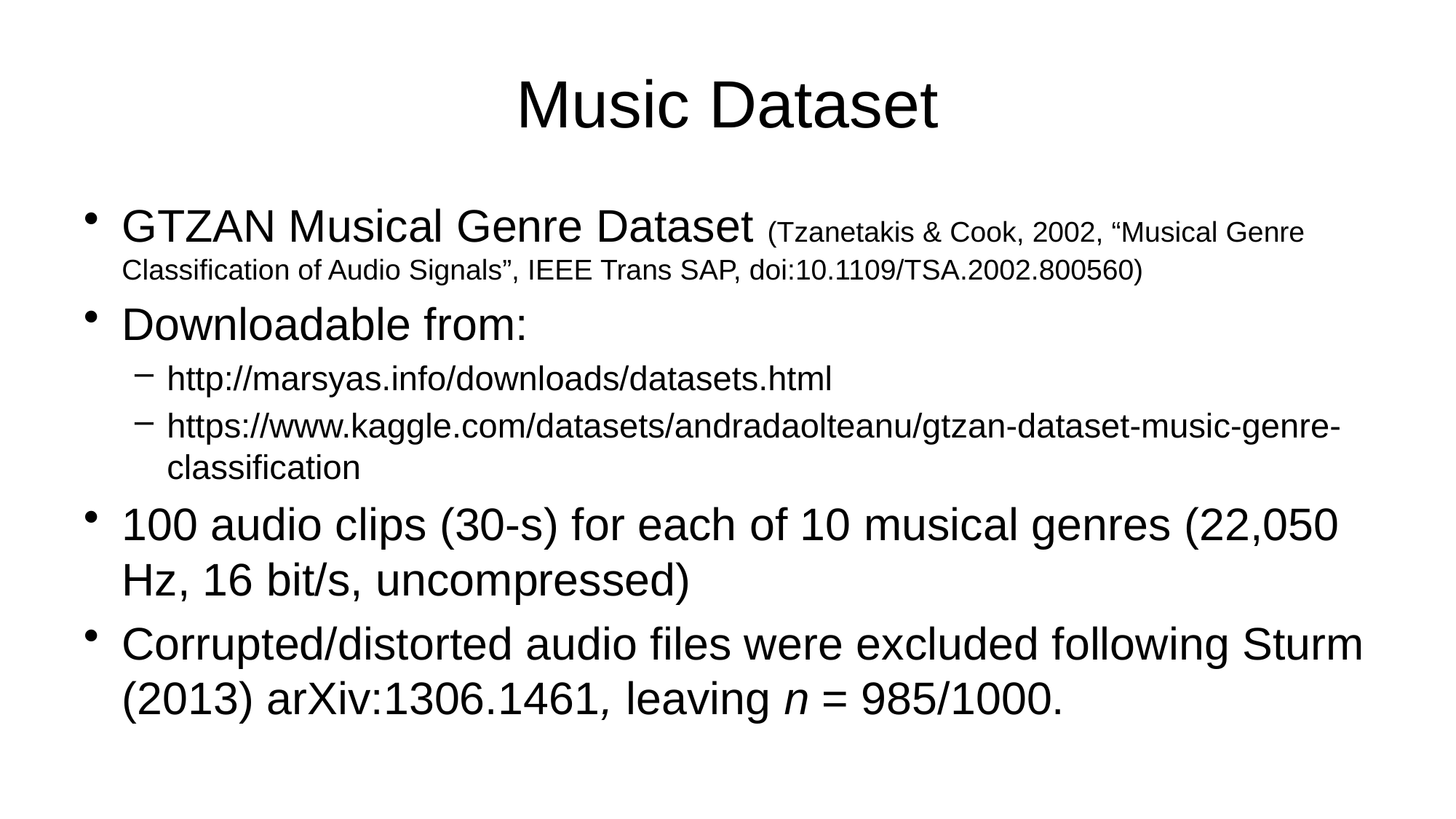

# Music Dataset
GTZAN Musical Genre Dataset (Tzanetakis & Cook, 2002, “Musical Genre Classification of Audio Signals”, IEEE Trans SAP, doi:10.1109/TSA.2002.800560)
Downloadable from:
http://marsyas.info/downloads/datasets.html
https://www.kaggle.com/datasets/andradaolteanu/gtzan-dataset-music-genre-classification
100 audio clips (30-s) for each of 10 musical genres (22,050 Hz, 16 bit/s, uncompressed)
Corrupted/distorted audio files were excluded following Sturm (2013) arXiv:1306.1461, leaving n = 985/1000.

## Slide 3
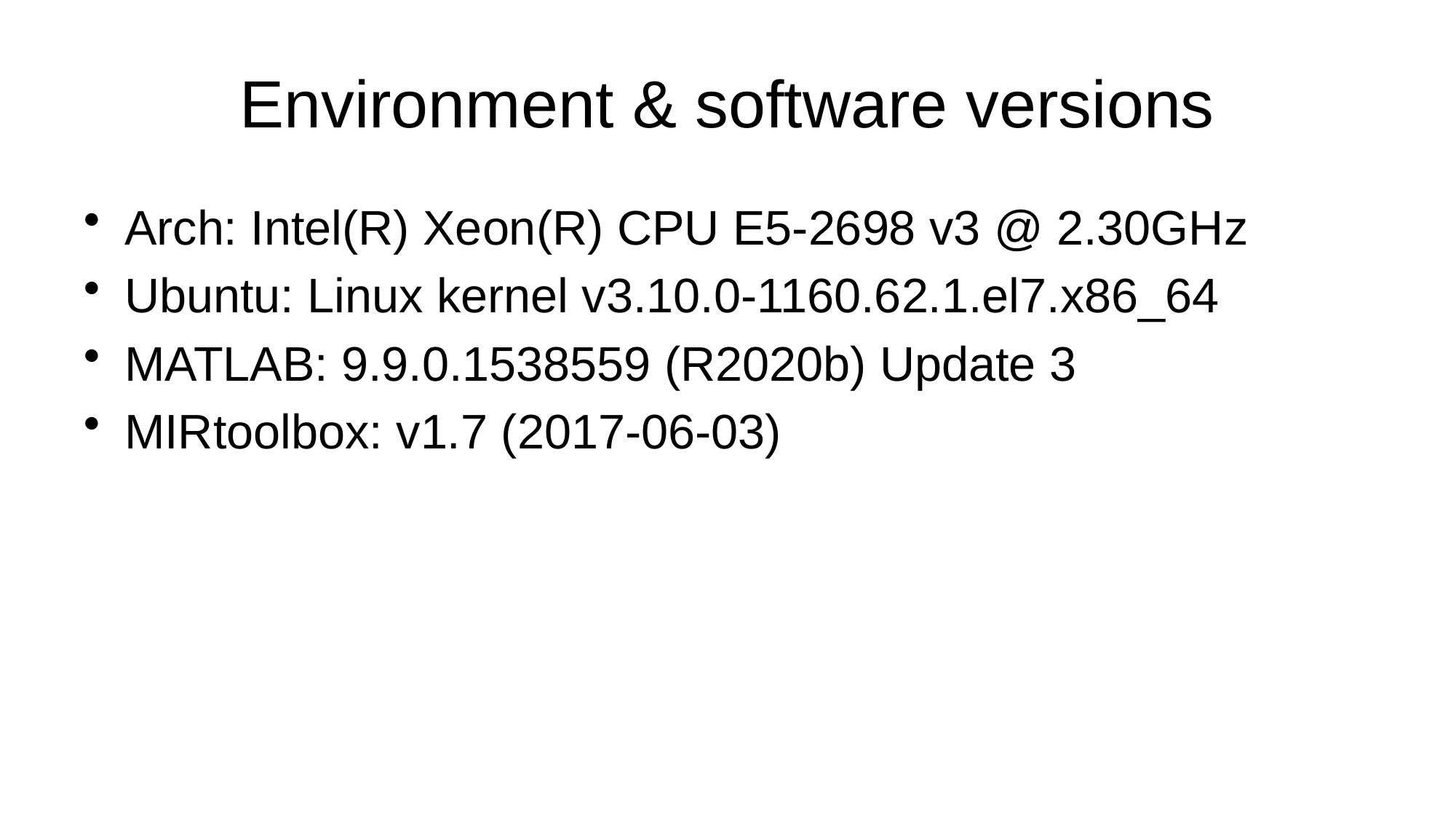

# Environment & software versions
Arch: Intel(R) Xeon(R) CPU E5-2698 v3 @ 2.30GHz
Ubuntu: Linux kernel v3.10.0-1160.62.1.el7.x86_64
MATLAB: 9.9.0.1538559 (R2020b) Update 3
MIRtoolbox: v1.7 (2017-06-03)

## Slide 4
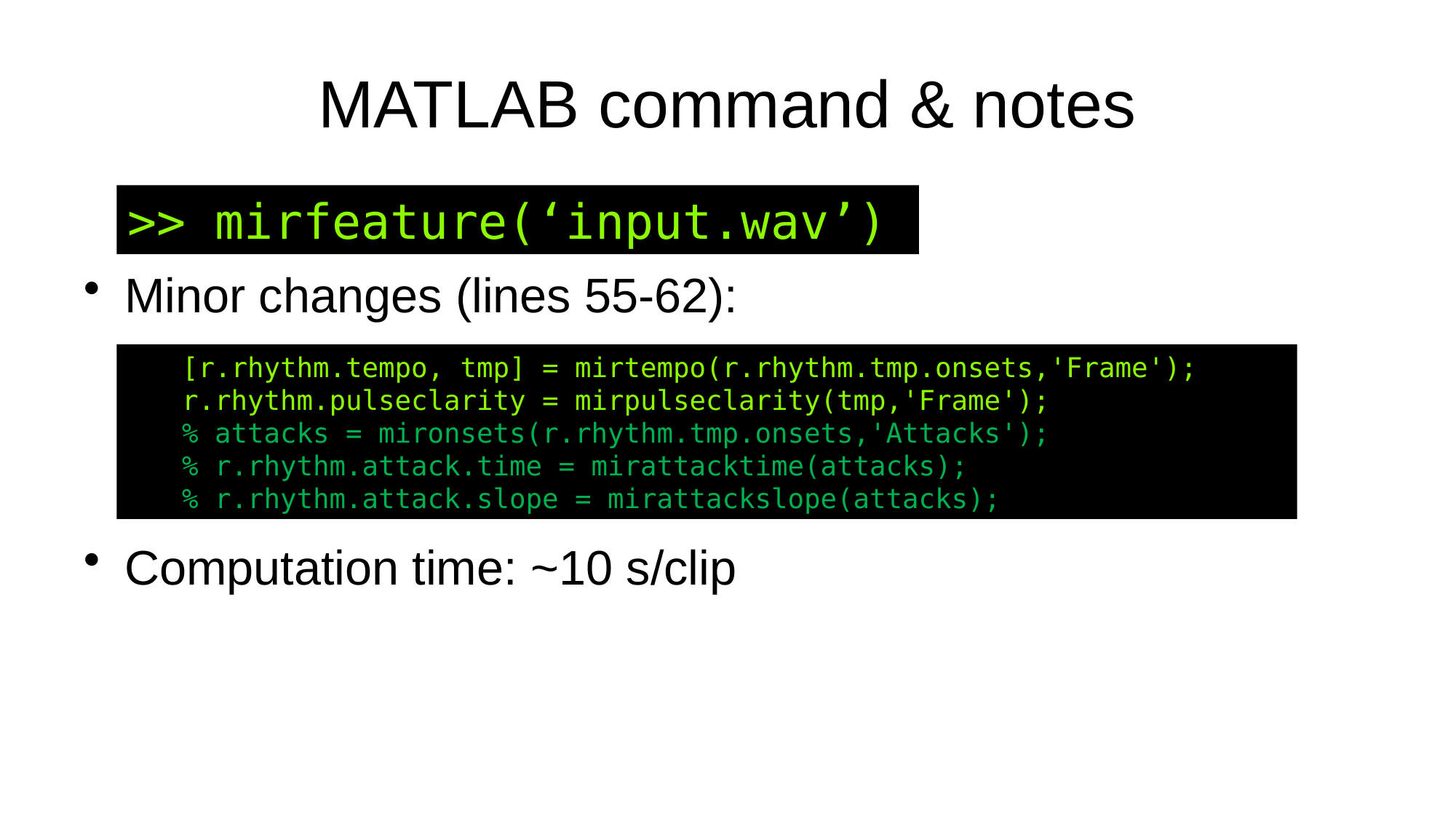

# MATLAB command & notes
>> mirfeature(‘input.wav’)
Minor changes (lines 55-62):
Computation time: ~10 s/clip
[r.rhythm.tempo, tmp] = mirtempo(r.rhythm.tmp.onsets,'Frame');
r.rhythm.pulseclarity = mirpulseclarity(tmp,'Frame');
% attacks = mironsets(r.rhythm.tmp.onsets,'Attacks');
% r.rhythm.attack.time = mirattacktime(attacks);
% r.rhythm.attack.slope = mirattackslope(attacks);

## Slide 5
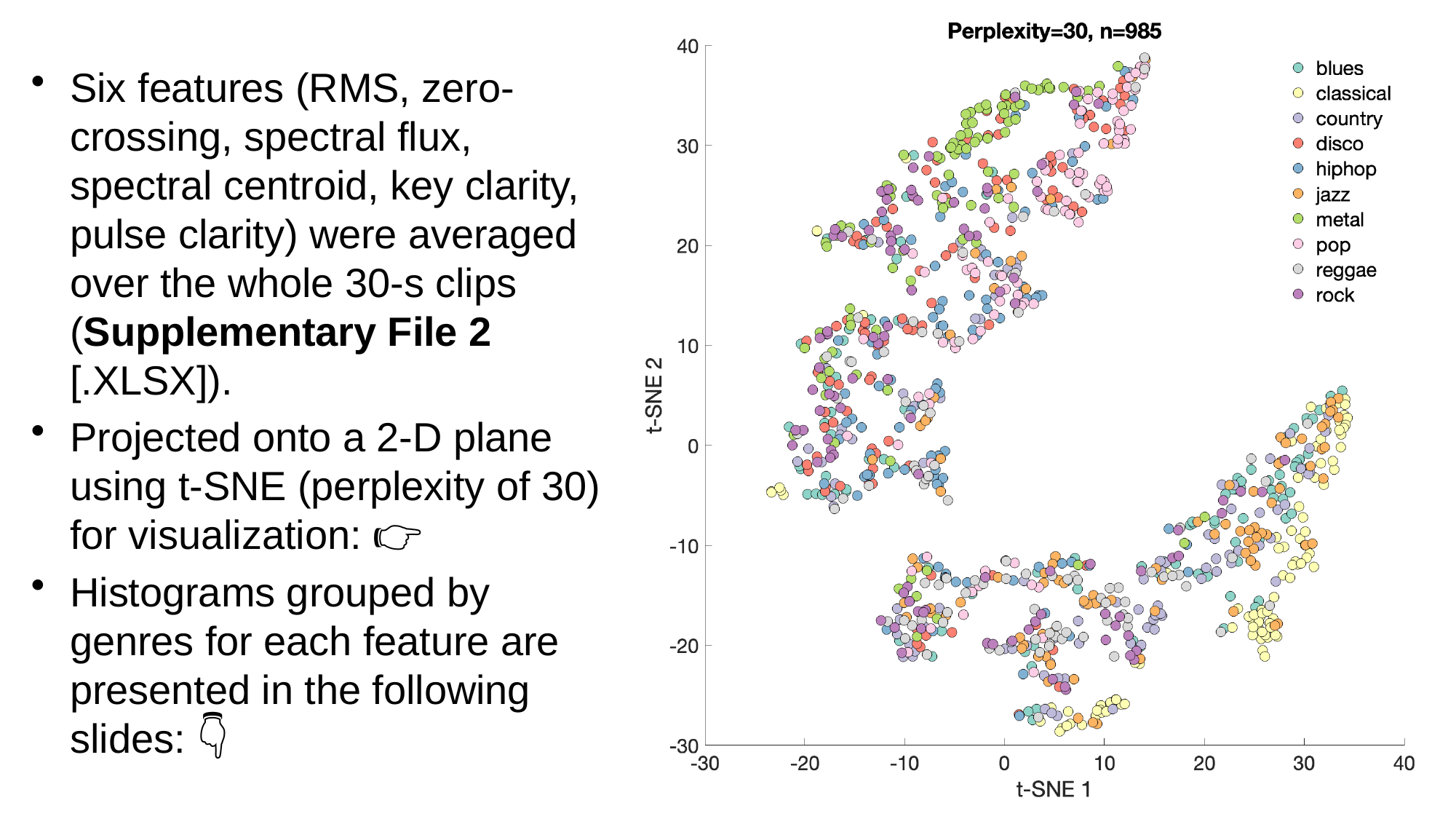

Six features (RMS, zero-crossing, spectral flux, spectral centroid, key clarity, pulse clarity) were averaged over the whole 30-s clips (Supplementary File 2 [.XLSX]).
Projected onto a 2-D plane using t-SNE (perplexity of 30) for visualization: 👉
Histograms grouped by genres for each feature are presented in the following slides: 👇

## Slide 6
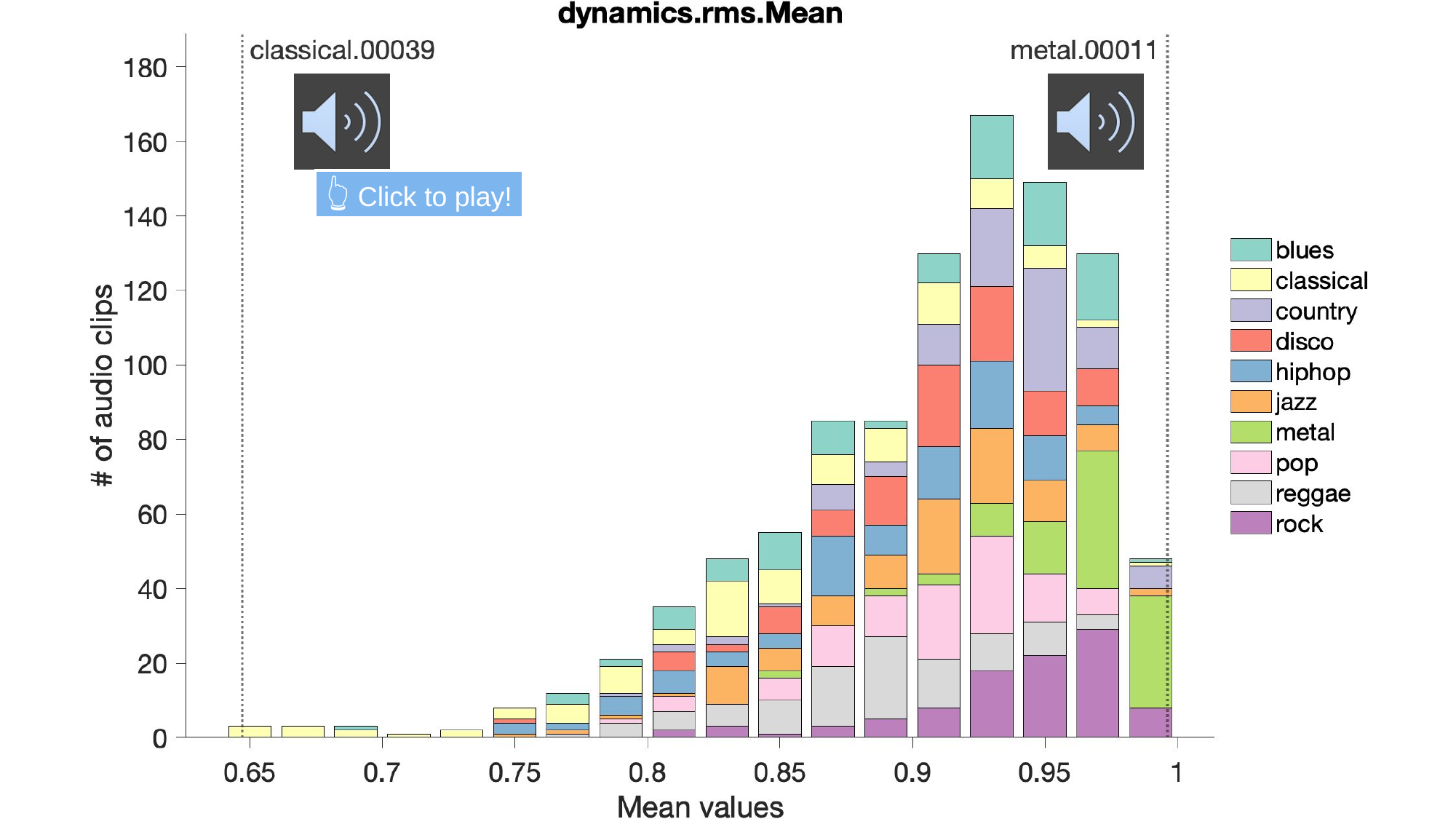

👆 Click to play!

## Slide 7
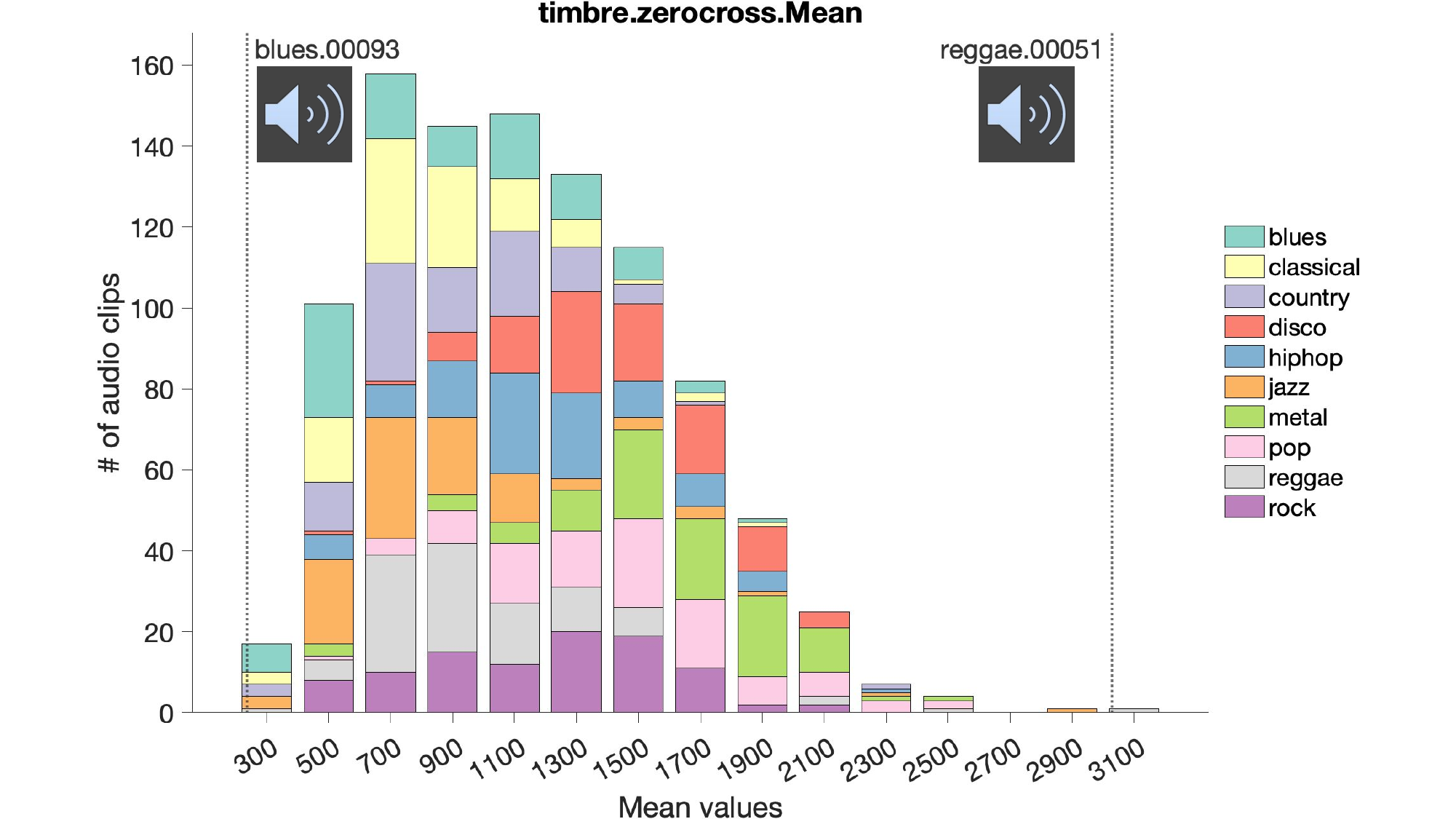

## Slide 8
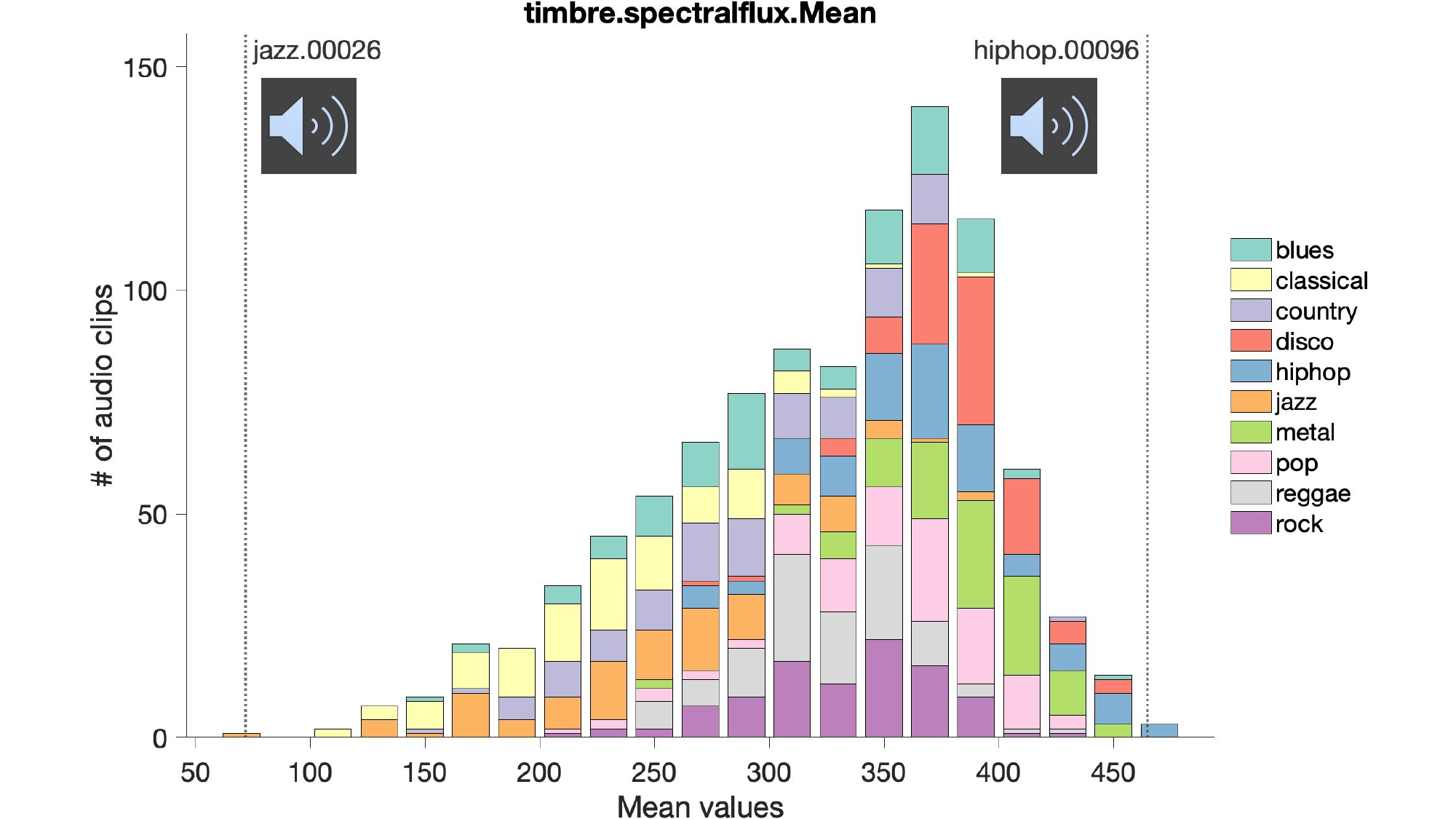

## Slide 9
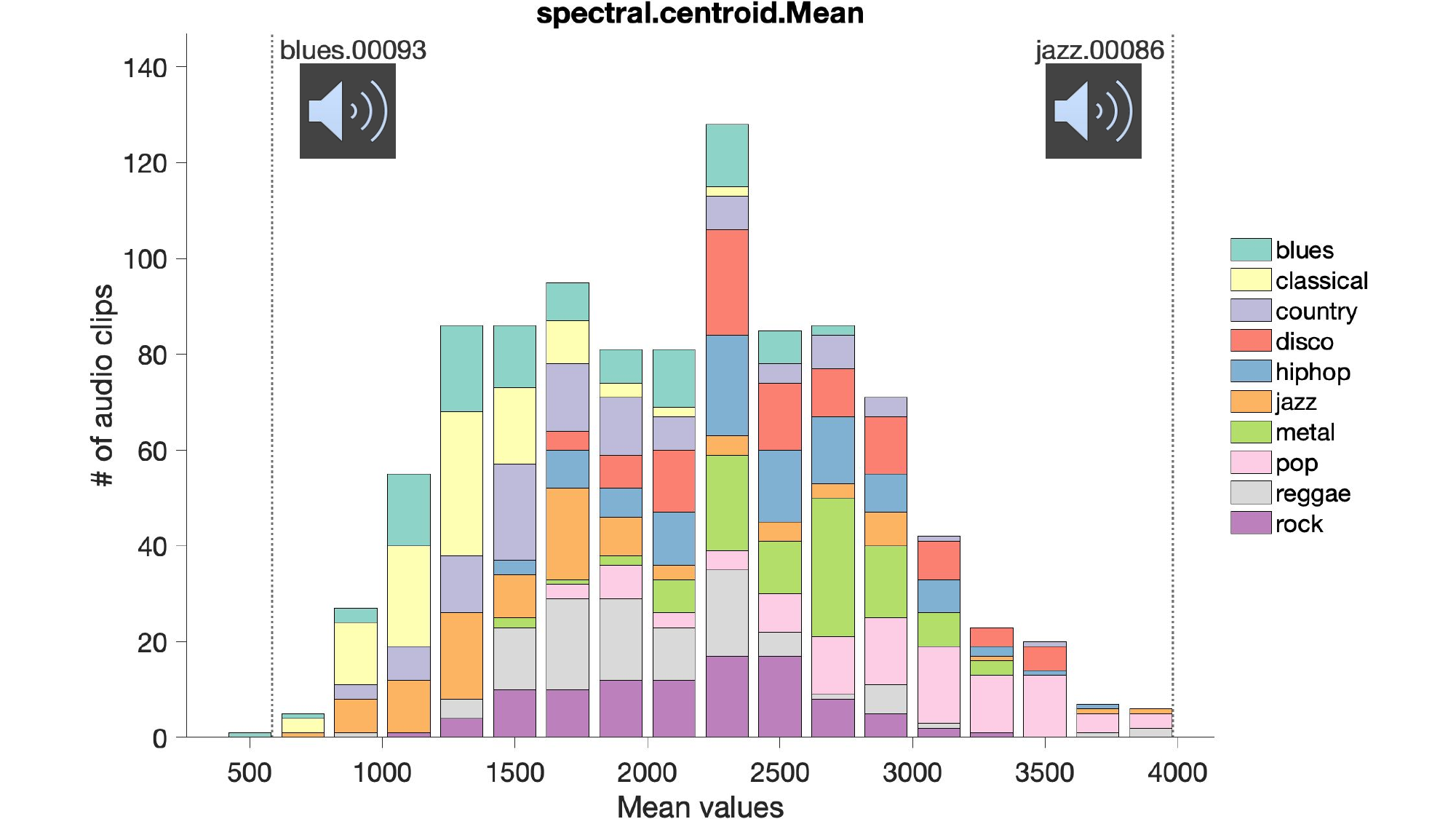

## Slide 10
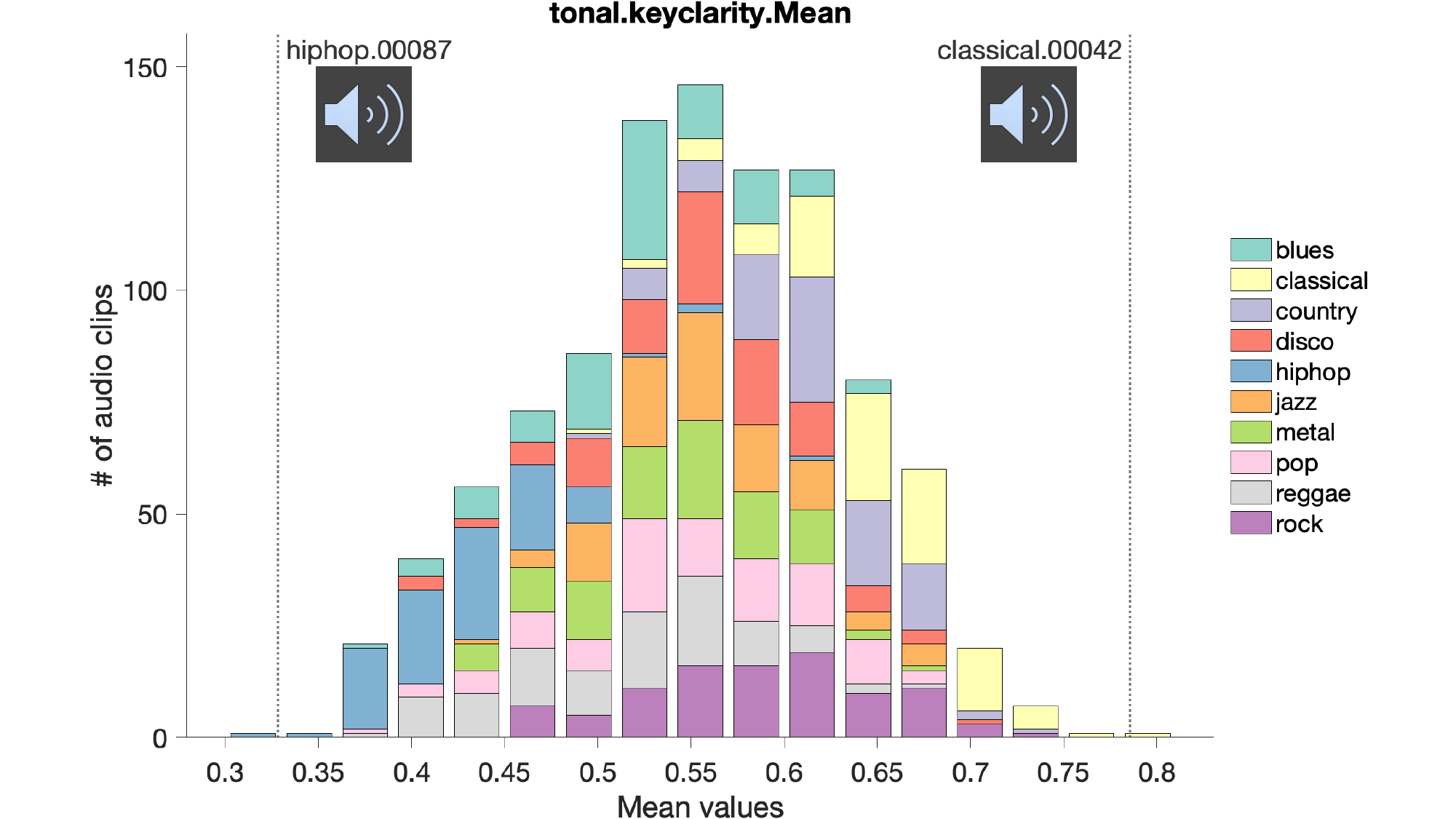

## Slide 11
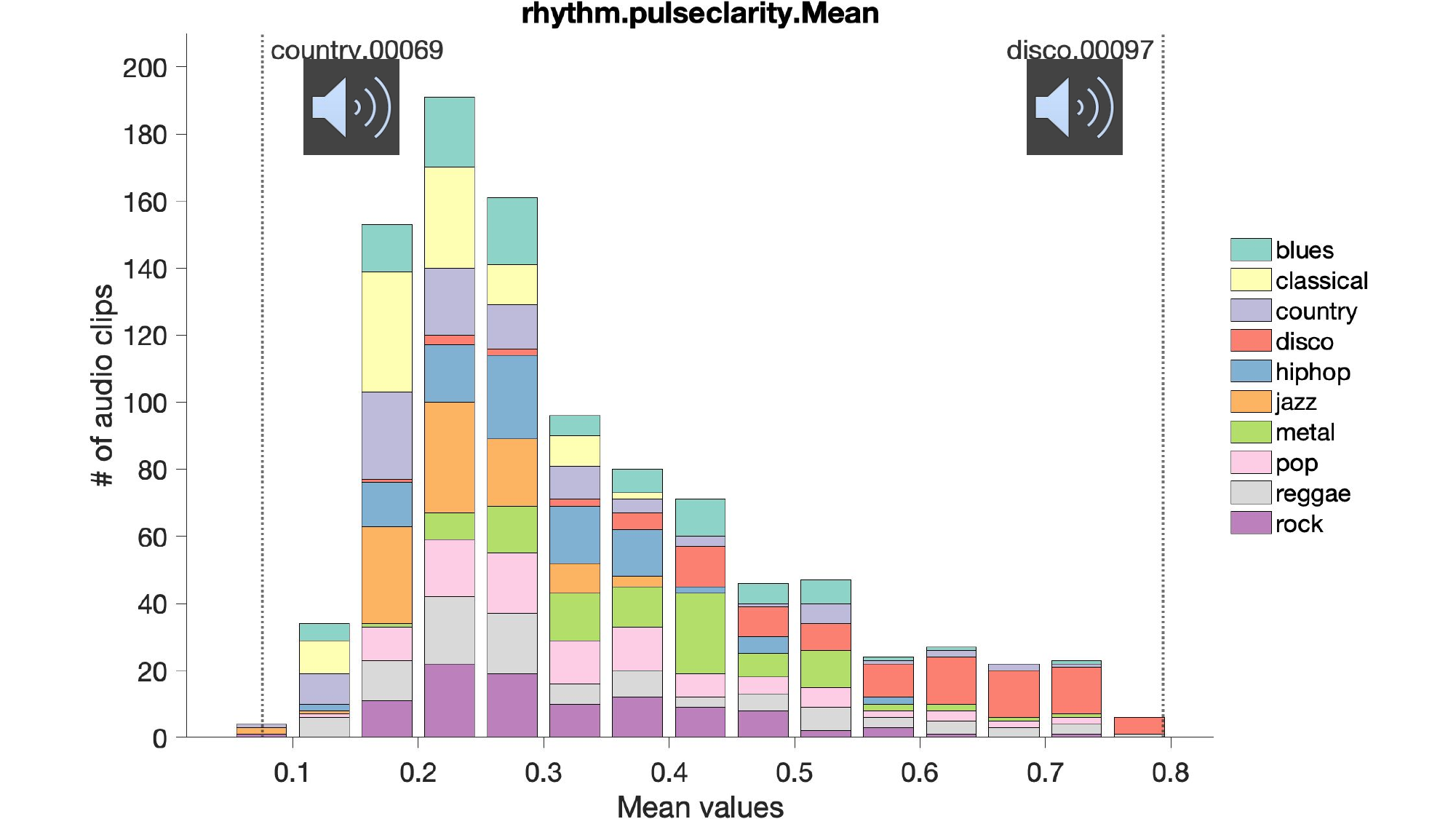

Supplement: Supplementary file 1 [file Presentation_1.PPTX]
